# Characterization of Multidrug Resistant *E*. *faecalis* Strains from Pigs of Local Origin by ADSRRS-Fingerprinting and MALDI -TOF MS; Evaluation of the Compatibility of Methods Employed for Multidrug Resistance Analysis

**DOI:** 10.1371/journal.pone.0171160

**Published:** 2017-01-30

**Authors:** Aneta Nowakiewicz, Grażyna Ziółkowska, Przemysław Zięba, Sebastian Gnat, Aleksandra Trościańczyk, Łukasz Adaszek

**Affiliations:** 1 Sub-Department of Veterinary Microbiology, Institute of Biological Bases of Animal Diseases, Faculty of Veterinary Medicine, University of Life Sciences, Lublin, Poland; 2 State Veterinary Laboratory, Lublin, Poland; 3 Department of Epizootiology and Clinic of Infectious Diseases, Faculty of Veterinary Medicine, University of Life Sciences, Lublin, Poland; Seconda Universita degli Studi di Napoli, ITALY

## Abstract

The aim of this study was to characterize multidrug resistant *E*. *faecalis* strains from pigs of local origin and to analyse the relationship between resistance and genotypic and proteomic profiles by amplification of DNA fragments surrounding rare restriction sites (ADSRRS-fingerprinting) and matrix-assisted laser desorption ionization time-of-flight mass spectrometry (MALDI -TOF MS). From the total pool of *Enterococcus* spp. isolated from 90 pigs, we selected 36 multidrug resistant *E*. *faecalis* strains, which represented three different phenotypic resistance profiles. Phenotypic resistance to tetracycline, macrolides, phenicols, and lincomycin and high-level resistance to aminoglycosides were confirmed by the occurrence of at least one corresponding resistance gene in each strain. Based on the analysis of the genotypic and phenotypic resistance of the strains tested, five distinct resistance profiles were generated. As a complement of this analysis, profiles of virulence genes were determined and these profiles corresponded to the phenotypic resistance profiles. The demonstration of resistance to a wide panel of antimicrobials by the strains tested in this study indicates the need of typing to determine the spread of resistance also at the local level. It seems that in the case of *E*. *faecalis*, type and scope of resistance strongly determines the genotypic pattern obtained with the ADSRRS-fingerprinting method. The ADSRRS-fingerprinting analysis showed consistency of the genetic profiles with the resistance profiles, while analysis of data with the use of the MALDI- TOF MS method did not demonstrate direct reproduction of the clustering pattern obtained with this method. Our observations were confirmed by statistical analysis (Simpson’s index of diversity, Rand and Wallace coefficients). Even though the MALDI -TOF MS method showed slightly higher discrimination power than ADSRRS-fingerprinting, only the latter method allowed reproduction of the clustering pattern of isolates based on phenotypic resistance and analysis of resistance and virulence genes (Wallace coefficient 1.0). This feature seems to be the most useful for epidemiological purposes and short-term analysis.

## Introduction

Bacteria from the genus *Enterococcus* constitute an important part of the intestinal biota both in humans and in animals. Among enterococcal species, mainly *E*. *faecium* and *E*. *faecalis* have the largest epidemiological importance since they are recognized as major nosocomial pathogens. From a medical perspective, the greatest risk associated with these species is the phenomenon of easily acquired resistance to many groups of antimicrobials, particularly to ampicillin, and high-level aminoglycoside and glycopeptide resistance, which significantly reduces the therapeutic alternatives and limits treatment to antimicrobials of last resort [1].

In the recent decades, it has frequently been demonstrated that food animals (including pigs) may constitute a reservoir of resistant bacteria and that the resistance genes may be easily transferred to the human population [1–3]. The phenomenon of spreading the resistance in *Enterococcus* among humans and animals is mainly due to transfer of conjugative and transferable elements carrying resistance genes during passage of enterococci of animal origin through the intestinal tract. Evidence of human infections caused by animal-origin enterococci has also been demonstrated and, although such cases are scarce, this path of transmission is possible [4].

Consequently, the analysis and differentiation of *Enterococcus*, primarily in terms of resistance, seems to have the greatest importance in epidemiological studies. The first step in this type of analysis is detection of the range and type of resistance, in which classical microbiological methods are still preferred to molecular techniques [5].

Many different methods have been used so far for typing *Enterococcus* bacteria in terms of their phenotypic and genotypic resistance [6–10]. However, most of the strains analyzed had a specific resistance profile, mainly including vancomycin- or high-level aminoglycoside-resistance, as groups with the greatest epidemiological significance. On the other hand, the increasing frequency of isolation of multidrug resistant strains from farm animals and food of animal origin enforces inclusion of strains resistant to other antimicrobials in epidemiological analysis and application of methods enabling reproducible typing of strains with a phenotypic resistance profile. Selection of appropriate methods for typing depends primarily on the type of research, especially the type of the problem to solve (local or broader epidemiological correlation and short- or long-term analysis) and the pool of strains tested (the size and degree of potential diversification). For analysis of the local diversity and short-term purposes, the typing method must have high discriminatory potential that will allow distinguishing very closely related isolates [11]. This criterion is met by the technique of ADSRRS-fingerprinting (amplification of DNA fragments surrounding rare restriction sites). Its discriminatory potential is comparable to the "gold standard" of PFGE [8] and using this method does not require prior knowledge of the sequences of the analysed strains. Moreover, this method is characterized by repeatability and ease of interpretation of results and has been successfully used for molecular typing of bacteria not only from the genus *Enterococcus*, [8,12], but also other bacterial species [13–16].

Rapid detection of resistance and capability of reproducible typing is another challenge for matrix-assisted laser desorption ionization time-of-flight mass spectrometry posed by researchers over the past few years [17,18]. As a tool for detection of antibiotic resistance and typing of different species of microorganisms, the MALDI -TOF MS technique has been described by many authors [9,19–23]. However, despite the high discriminatory potential, the results are highly diverse, depending on the species of the microorganism and the pool of strains tested. Still, it has not been conclusively confirmed whether this method is a suitable tool for comparative analysis of strains with varying phenotypic resistance profiles.

Local resistance analysis or analysis of closely related strains requires the use of methods with high discriminatory potential. On the other hand, it is desirable that its results should facilitate demonstration of the potential relationship between the resistance pattern, genotype, or protein expression pattern to allow tracking the mode and direction of the spread of multidrug resistant strains in animals and humans.

The aim of this study was to carry out epidemiological analysis of multidrug resistant *E*. *faecalis* strains from pigs and to determine the relationship between phenotypic resistance and genotypic and proteomic profiles obtained by ADSRRS-fingerprinting and MALDI- TOF MS methods, respectively.

## Material and Methods

### Sampling and species identification

The samples were collected from April to June 2015 in three closed-cycle intensive pig farms (designated as 1,2 and 3) in eastern Poland, spaced at least 30 km apart. All the farms are in Lublin province, and are distributed according to the following geographical coordinates: the farm No 1—51°20′39″N 22°24′45″E, Bychawa commune, district of Lublin; the farm No 2—51°29′28″N 22°51′18″E Ostrów Lubelski commune; district of Lubartów; the farm No 3—51°45′N 22°47′E Wohyń commune, district of Radzyń Podlaski

The material for the study was collected from farms where the presence of multi-drug resistant strains of *E*. *faecalis* was previously demonstrated in animals [24]. Since material came from a species not covered by the legal protection of species (farm animals) the consent of the ethics committee was not required. Swabs were taken by veterinary surgeon in the course of routine clinical examinations as part of control of animals health with the approval of owners of farms.

On all farms, the pigs were housed indoors on deep litter (straw). The following information concerning the use of antibiotics for treatment and metaphylaxis during past few years on these farms was available: penicillin, oxytetracycline, enrofloxacin, florfenicol, and streptomycin were used on all farms, in addition to lincomycin, spectinomycin, tiamulin, and tylosin on farms 1 and 3.

The material for the study consisted of rectal swabs from 90 randomly selected clinically healthy animals (30 animals of each farm). The samples were collected from the rectum using a sterile cotton swab. The swabs were incubated in buffered peptone water for 12 h at 37°C; then the material was plated (100 μl) on Slanetz-Bartley Agar (Biocorp, Warsaw, Poland) and incubated at 41°C for 48 h. Four most macroscopically different colonies from each sample were chosen for further analysis.

Identification to the genus *Enterococcus* was performed as previously described [12]. Species identification was performed using 16S–23S rRNA intergenic region restriction endonuclease analysis according to previously described protocols [25].

### Antimicrobial susceptibility testing

Drug susceptibility of the isolates was evaluated using the microdilution method (house kits prepared by ourselves were used) in accordance with the standards of the Clinical and Laboratory Standards Institute [26] (*E*. *faecalis* ATCC 29212 and *E*. *faecalis* ATCC 51299 were used as quality controls). The phenotypic resistance profiles were established according to breakpoints (S1 Table) for the following antimicrobials: ampicillin, chloramphenicol, ciprofloxacin, enrofloxacin, erythromycin, gentamicin, kanamycin, lincomycin, quinpristin-dalphopristin, rifampin, streptomycin, tetracycline, tylosin, and vancomycin. Multidrug resistance was defined as a profile including resistance to at least one agent in three or more antimicrobial classes [27].

### Genotypic analysis

DNA was isolated using ready-made kits (Bacterial and Yeast Genomic DNA Purification Kit; Eurx, Gdańsk, Poland) according to protocols described elsewhere [12]. The isolates were tested for the presence of selected genes encoding resistance to macrolides [*erm*A, *erm*B, *erm*C, *erm*F, *msr*A, *mef*A], tetracycline [*tet*M, *tet*K, *tet*O,*tet*L, *tet*S], phenicols [*cfr*, *fex*A, *cat*], lincosamides [*lnu*B, *lnu*F], copper [*tcr*B] as well as aminoglycosides and aminocyclitols [*aac*(6’)-Ie-*aph* (2”)-*Ia*, *aph*(3’)-*IIIa*, *aph*(2”)-*Ib*, *aph*(2”)-*Ic*, *aph*(2”)-*Id*, *ant*(6)-*Ia*, *ant*(4)-*Ia*, *ant*(9)-*Ia*, and *aad*A], and genes encoding virulence factors: *gel*E (gelatinase), *cyl*A, *cyl*B, *cyl*M (hemolysin-cytolysin production), *efa* Afs (cell wall adhesin), *agg* (aggregation protein), *esp* (cell wall-associated protein involved in immune evasion), *hyl* (hyaluronidase) *cpd*, *cob*, and *ccf* (sex pheromones facilitate conjugation). The PCR assay was also used to demonstrate the presence of the Tn*5397*-like (resolvase gene *tnd*X) and *Tn916/Tn1545*-like (integrase gene *Int-Tn*) transposons.

The primers and cycling conditions used for detecting resistance and virulence genes were as previously published (S2 Table). All reactions were performed in a thermal cycler (T Personal thermal cycler–Biometra GmbH, Goettingen, Germany), using Gold Taq MIX (Syngen Biotech, Wrocław, Poland) and appropriate primers (Sigma Aldrich, Germany).

Amplification of DNA fragments surrounding rare restriction sites (ADSRRS- fingerprinting) was performed as described by Krawczyk et al. [8] with a few modifications [12]. The reaction of restriction endonuclease digestion of genomic DNA (150–250ng) was carried out with 25 μl of a reaction mixture composed of 10 U *Xba*I, 5 U *Bgl*II, and 5 μl Tango buffer (ThermoScientific^®^, Waltham, USA) at 37°C, for 60 min (the reaction was stopped by thermal inactivation at 80°C for 2 min). For ligation, appropriate adapters [8], corresponding to cohesive ends of restriction fragments and T4 Ligase (0.5μl) were used. The ligation reactions were carried out at room temperature for 60 min and then thermal inactivation at 70°C for 5 min was performed. The PCR reaction was carried out using 25 μl of a reaction mixture composed of 2 μl of the solution after the ligation reaction, 5 μl of Gold Taq MIX (Syngen Biotech, Wrocław, Poland) and 50 pmol of each primer (Genomed, Warsaw, Poland).

The reaction conditions for the thermal cycler (T Personal thermal cycler–Biometra GmbH, Goettingen, Germany) were as follows: initial cycle of 94°C for 5 min, 72°C for 5 min to fill in the ends of the DNA fragments, and initial denaturation at 94°C for 5 min (pre-PCR), followed by 22 cycles of 94°C for 30 s, 56°C for 30 s, and 72°C for 1.5 min, after which an extension cycle of 72°C for 5 min was added. Electrophoretic separation of PCR products was carried out in 6% polyacrylamide gel (Sigma-Aldrich Germany). Electrophoretic profiles were fixed using Vision-capt Quantum (Vilber Lourmat, France).

BIO-1D++ 11.9 software (Vilber Lourmat, France) was used for cluster analysis of the strains by the Unweighted Pair Group Method with Arithmetic Mean (UPGMA). The similarity index of the isolates was calculated using the Dice correlation coefficient option of the software with position tolerance and optimization of 1%.

### Matrix-assisted laser desorption/ionization time-of-flight mass spectrometry (MALDI-TOF MS) experiments

Bacteria were prepared for mass spectrometry analysis according to a standard extraction protocol using formic acid, as recommended by the Bruker Company (OP for Sample Preparation Using Formic Acid Extraction Method). A volume of 0.5 μl of the prepared material (full extraction of bacterial proteins) was applied to an MTP 384 Ground Steel target (BrukerDaltonics). An HCCA (α-Cyano-4-hydroxycinnamic acid) matrix solution (suspended in a standard solution recommended by the manufacturer, BrukerDaltonics, GmbH Bremen Germany) was placed on each bacterial sample. The recommended BrukerDaltonics Bacterial Test Standard (BTS) was applied in a configuration such that one location of the standard was positioned at the centre of 4 bacterial sample locations. External calibration was performed using a standard calibration mixture of an *E*. *coli* extract (BrukerDaltonics GmbH Bremen, Germany) containing RNase A and myoglobin proteins. The bacterial spectra were acquired using an ultrafleXtreme mass spectrometer from Bruker Daltonics (FlexControl 3.0 software), and then analysed using Bruker BioTyper 3.0. The spectrum of the bacteria was obtained in a positive linear mode, using an acceleration voltage of 24 and 23 kV. The spectra were collected within a mass range of 2 to 20 kDa. The analysis was repeated for three separate cultivations of each strain and three times for each sample. In the first step of the spectral analysis, the commercial BioTyper database containing 5672 entries (Jan 2016) was used for species identification. The spectra were preprocessed by smoothing with the Savitzky-Golay method. The baseline correction was performed using the Top-Hat baseline algorithm and peak geometry was characterized by means of the Standford Network Analysis Platform (SNAP) algorithm. Only peaks that had a three times higher signal-to-noise ratio were considered. The spectra obtained were manually processed using FlexAnalysis software (ver. 3.3) and peak mass lists were created. Based on the principle that the identification score reflects compatibility of the spectra with the standard strains from the reference database, the main spectrum (MSP) profile showing the highest value of log (score) (the most representative for each isolate) was selected for further analysis. For visualization of enterococci, the pseudo gel view was produced from mass spectra (intensities are gray-scaled log scale).

### The statistical comparative analysis

All data were recorded in an Excel file, including the peak mass lists generated by flexAnalysis 3.3 and converted in NTSYSpc ver. 2.0 software (Exeter Software, NY, USA). For cluster analysis (dendrograms), the Unweighted Pair Group Method with Arithmetic Mean (UPGMA) with the Dice coefficient was used.

In order to compare the discriminatory potential of the analysis of phenotypic resistance in relation to the analysis of the gene profile (both resistance and virulence), genome (ADSRRS-fingerprinting) and protein profile (MALDI-TOF MS), Simpson's index of diversity (SID) with 95% confidence intervals (95% CI) was calculated. The function of this index is numerical evaluation of the possibility of assignment two randomly selected strains to separate groups, i.e. their diversity. Compatibility between the groupings of strains with the different techniques was determined based on the Rand coefficient and Wallace coefficient.

The first of these coefficients shows the absolute value of the compatibility of methods, compared in pairs, adjusted for the randomized case of their full compliance. The resulting matrix of the results is symmetrical. In turn, the bidirectional Wallace coefficient indicates the degree of overlap between the groups of analyzed strains obtained with other methods, which allows determining whether two strains classified together in one method will also be grouped together in another method. In this way, we determine the probability of generation of a corresponding dendrogram. The Wallace coefficient is designed to predict to what extent the grouping obtained with one technique can be used to determine the outcome of another method. It also predicts whether the use of any of the methods for determination of the diversity of strains is redundant.

All analyses were performed in the EpiCompare software (Ridom Bioinformatics, Munster, Germany)

## Results

In total, 321 isolates belonging to the genus *Enterococcus* were obtained, with 40% (128 strains) classified as *E*. *faecalis* (16S-23S rRNA REA). Using the MIC value, seven distinct phenotypic resistance profiles were separated in the pool of the isolates tested (data not shown).

Thirty six resistant strains of *E*. *faecalis* belonging to three different phenotypic resistance profiles (A,B and C) met the criteria of multidrug resistance and these strains were selected for further studies (each selected strain came from another animal) (S1 Table).

Profiles A and B were homogeneous in terms of the source (the strains were isolated from animals originating from farms 1 and 3 respectively) but isolates from profile C originated from animals from both farms 2 and 3.

All selected strains were susceptible to ampicillin and vancomycin and resistant to tetracycline, chloramphenicol, linkomycin, high-level kanamycin and streptomycin, as well as macrolides (erythromycin and tylosin) and glycopeptides (quinpristin-dalphopristin). The resistance to other antimicrobials varied between the three profiles. Strains belonging to profile A were resistant to all other antimicrobials tested with the exception of rifampin. Strains from profile B were susceptible to rifampin and high-level gentamycin, and strains of profile C exhibited resistance to rifampicin and susceptibility to both fluoroquinolones (S1 Table). Phenotypic resistance to tetracycline, macrolides, phenicols, and lincomycin and high-level aminoglycoside resistance to kanamycin, streptomycin, and gentamycin were confirmed by occurrence of at least one corresponding resistance gene in each strain belonging to a particular profile.

The presence of 12 genes of all the 28 resistance genes and 2 transposone genes (resolvase and integrase) tested was shown (Table 1). All the strains showed the presence of *tet*M, *erm*B, *aph(3’)-IIIa*, and *ant(6)-Ia*. The distribution of the other genes varied and correlated with the phenotypic resistance profile. The following resistant genes: *cat*A-8, *lnu*B, *Int-Tn*, and *aac(6’)-Ie-aph(2”)-Ia*, were further detected in strains belonging to profiles A and C, while the genotypic resistance profile of the strains belonging to group B was characterized by the presence of the *fex*A gene. Furthermore, the strains belonging to both profiles A and B had the *tet*L gene.

**Table 1:** Occurrence of resistance and virulence genes in three selected phenotypic resistance profiles of *E*. *faecalis*.

phenotypic resistance profiles /No of strains	resistance gene profile/No of strains	resistance and transposon genes	virulence genes
profile A/9 CFMGKLQDST	I/5	*Int-Tn*, *aac(6’)-Ie-aph(2”)-Ia*, *aph(3’)-IIIa*, *ant(6)-Ia*, *erm*(B), *tet*(M), *tet*(L), *cat*A8, *lnu*B	*agg*, *esp*, *cpd*, *cob*, *ccf*, *efa*Afs
	II/4	*Int-Tn*, *aac(6’)-Ie-aph(2”)-Ia*, *aph(3’)-IIIa*, *ant(6)-Ia*, *erm*(B), ^a^***erm*(F)**, *tet*(M), *tet*(L), *cat*A8, *lnu*B	
profile B/13 CFMKLQDST	III/13	*aph(3’)-IIIa*, *ant(6)-Ia*, *erm*(B), *tet*(M), *tet*(L), *fex*A	*gel*E, *cpd*, *cob*, *ccf*, *efa*Afs
profile C/14 CMGKLQDRST	IV/8	*Int-Tn*, *aac(6’)-Ie-aph(2”)-Ia*, *aph(3’)-IIIa*, *ant(6)-Ia*, *erm*(B), *tet*(M), *cat*A8, *lnu*B	*agg*, *esp*, *cyl*A, *cyl*B, *cyl*M, *cpd*, *cob*, *ccf*, *efa*Afs
	V/6	*Int-Tn*, *aac(6’)-Ie-aph(2”)-Ia*, *aph(3’)-IIIa*, *ant(6)-Ia*, *erm*(B), *tet*(M), *cat*A8, ***cat*A9**, *lnu*B	

^a^boldface indicates differentially expressed genes within each phenotypic resistance profiles.

Nearly half of the strains from profile A (44,4%) had the *erm*F gene and 42,9% of the strains from profile C had the *cat*A-9 gene (wherein all *cat*A-9 positive isolates originated from animals of farm No 2) (S1 Table).

Based on the analysis of genotypic and phenotypic resistance in the group of multidrug resistant *E*. *faecalis*, five distinct genotypic resistance profiles were generated (Table 1).

The profiles of virulence genes were less diverse than the genetic resistance profiles; however, they included at least five different genes. Only three distinct genotypic virulence profiles were generated and these profiles closely corresponded to the phenotypic resistance profiles (Table 1). From the eleven virulence genes tested, only the *hyl* gene (encoding hyaluronidase) was not demonstrated in any of the strains analyzed. Genes encoding sex pheromones (*cpd*, *cob*, *ccf)* and *efa*A*fs* were present in all the strains tested. The *gel*E gene was present in strains from profile B and the two other virulence genes *(agg*, *esp*) were detected in all the strains belonging to profile A and C; additionally, the presence of the *cyl* gene was noted in the latter profile.

**Fig 1:**
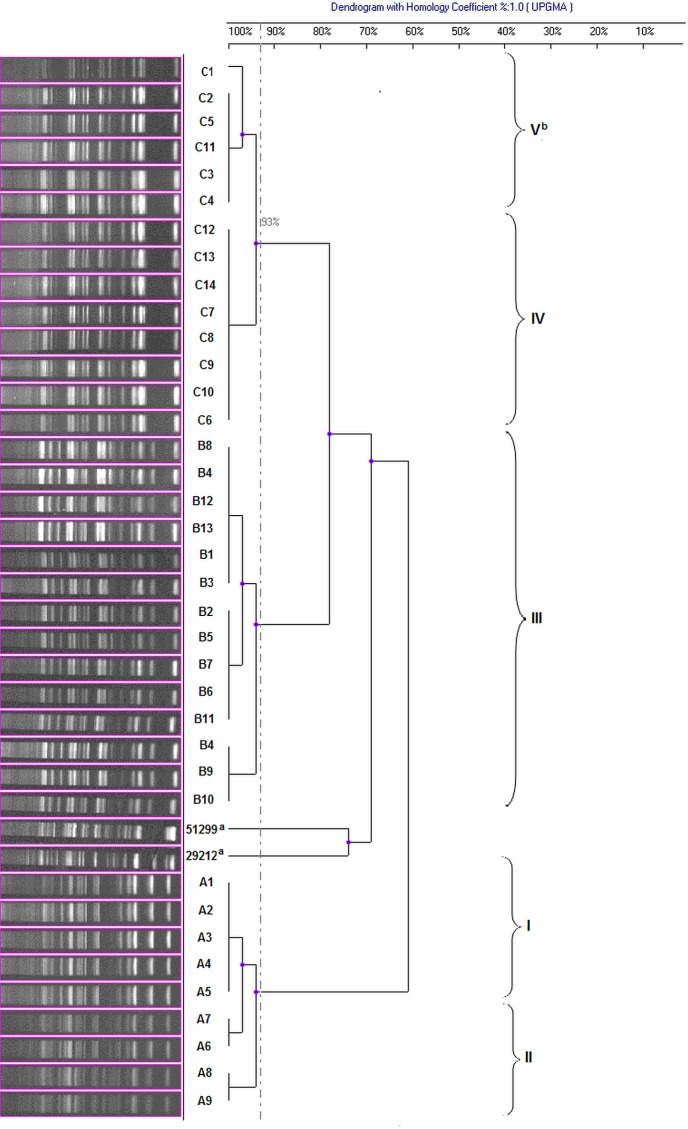
Dendrogram of genetic relatedness among *E*. *faecalis* strains obtained with the ADSRRS-fingerprinting method. ^a^ reference strains of *E*. *faecalis*: ATCC29212 and ATCC51299 ^b^ resistance gene profile.

The genotypic ADSRRS-fingerprinting analysis revealed the presence of 18 to 22 bands ranging in size from 110 to 1.500 bp (Fig 1). The strains were grouped in three major clusters (with the exception of the reference strains) corresponding to the phenotypic resistance profiles (Fig 1). The similarity coefficients between the groups clustering the *E*. *faecalis* strains belonging to the three phenotypic profiles were 0.78 for profiles C and B and 0.7 for profile A, compared to the other strains. The percentage of similarity of the strains was considerably higher within the major groups and fluctuated around 93% in each group.

The spectral analysis using the BioTyper software and BioTyper database confirmed that all of the examined mass spectra of *Enterococcus* tested were assigned to correct species with an average score value of 2,1 (including all repeated spectra) validating probable species identification according to the Bruker guide.

**Fig 2:**
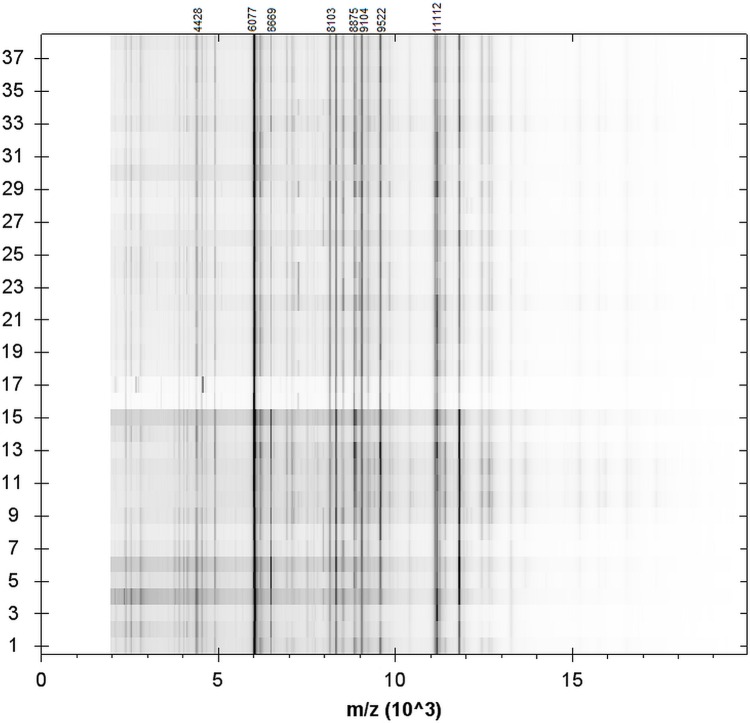
Pseudo gel view representation obtained from the average mass spectra of *E*. *faecalis*.

Only representative spectra from each strain with the highest log (score) were assigned for further analysis (the average log(score) was 2,344; CI: 2,354–2,334). In total, 189 distinct peaks were obtained by MALDI TOF MS, with 25 present in all the strains tested (S3 Table). In the next stage of our analysis, we examined the pseudo gel view (in the mass range between m/z 3000–13000), which converts spectral peak intensities to gray-scales (Fig 2). Among the most intense mass peaks, we found 9 peaks occurring in all the strains: 11112 m/z, 9522 m/z, 9104 m/z, 8875 m/z, 8103 m/z, 6669 m/z, 6223 m/z, 6077 m/z, and 4428 m/z (Fig 2).

### Comparative analysis

**Fig 3:**
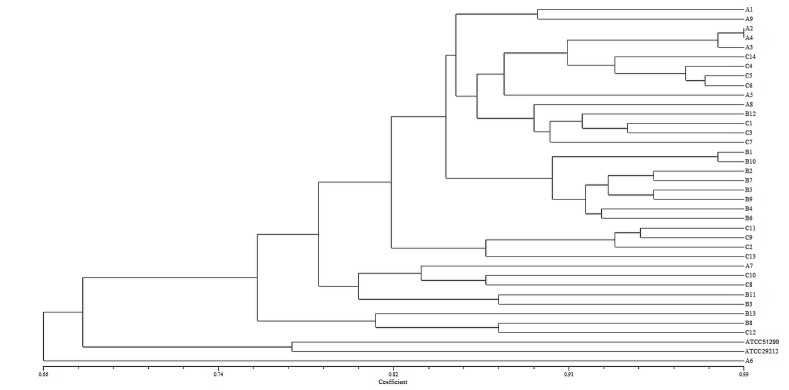
Dendrogram obtained after NTSYSpc analysis of MALDI-TOF MS data.

To compare the discriminatory power and the congruence between the type assignments of the different typing methods (Figs 1 and 3, S1 and S2 Figs), Simpson’s index of diversity and an adjusted Rand and Wallace coefficient were calculated for 36 isolates for which the results of typing methods were available (Table 2).

**Table 2:** Statistic comparison of the typing methods used in this study.

Technique	Simpson’s index of diversity (95% CI)	Rand coefficient (95% CI)			Wallace coefficient (95% CI)			
		MALDI TOF MS	Phenotype	Genotype	MALDI TOF MS	Phenotype	Genotype	ADSSRS
MALDI TOF MS	0.941 (0.899–0.983)					0.595 (0.434–0.755)	0.243 (0.049–0.437)	0.162 (0.00–0.263)
Phenotype^a^	0.386 (0.241–0.530)	0.397 (0.256–0.538)			0.057 (0.011–0.103)		0.354 (0.263–0.445)	0.082 (0.008–0.157)
Genotype^b^	0.783 (0.717–0.848)	0.752 (0.673–0.832)	0.603 (0.483–0.723)		0.066 (0.00–0.140)	1.0		0.472 (0.371–0.572)
ADSSRS- fingerprinting	0.884 (0.848–0.920)	0.844 (0.790–0.899)	0.502 (0.359–0.645)	0.898 (0.822–0.975)	0.082 (0.00–0.196)	1.0	1.0	

^a^ phenotype indicates the phenotypic resistance profile.

^b^ genotype indicates the profiles of resistance and virulence genes.

Simpson's index of diversity was in the range of 0.386 (0.241–0.530) for the analysis of phenotypic resistance, 0.783 (0.717–0.848) for the analysis of resistance and virulence genes, and 0.884 (0.848–0.920) for the ADSRRS-fingerprinting technique. It had the highest value of 0.941 (0.899–0.983) for MALDI-TOF MS. Maximum values of the Rand coefficient (showing good correlations between the techniques) amounting to 0.898 (CI 0.822–0.975) and 0.844 (CI 0.790–0.920) were obtained while comparing ADSRRS-fingerprinting with the analysis of genes and

ADSRRS-fingerprinting with MALDI -TOF MS, respectively. In contrast, the MALDI-TOF MS analysis and phenotypic resistance exhibited the lowest compliance of clustering; in this case, the Rand coefficient was only 0.397 (CI 0.256–0.538). Application of the ADSSRS-fingerprinting method allows accurate projection of clustering obtained by analysis of the phenotypic resistance profile and analysis of gene profiles (Wallace coefficient 1.0). However, it significantly deviates from the clustering of strains by MALDI-TOF MS technique (0.082). On the other hand, the latter technique can predict the outcome of the typing method of phenotypic resistance testing at the highest level (0.595), which is substantially higher than in the case of the analysis of the gene profile and ADSRRS-fingerprinting (respectively 0.243 and 0.162). In addition, the analysis of the occurrence of the genes in the strains tested can accurately reproduce the grouping obtained in the phenotypic resistance analysis (1.0); symmetrically, the compliance result is much weaker (0.354). In the other cases, the value of the Wallace coefficient had a relatively low value, i.e. in the range of 0.057–0.082.

## Discussion

The prevalence of antimicrobial resistance in commensal gut bacteria including *Enterococcus* is a good indicator of the selective pressure caused by the use of antimicrobials in farm animals. *Enterococcus* bacteria constitute a significant proportion of the gastrointestinal part of animal microbiota and fecal contamination of carcasses during slaughter processes [3,28]. Direct and indirect contact with animals may favor the spread of bacteria. Enterococci originating from animals (including pigs) are also considered as an important reservoir of resistance genes, which may be transferred to other human pathogens [28–31]. Special attention should be paid to multidrug resistant strains of *Enterococcus*, and especially those belonging to the species *E*. *faecalis*, which is the third most prevalent nosocomial pathogen worldwide [32]. Therefore, multidrug resistant (MDR) *E*. *faecalis* strains isolated from farm animals were chosen for our study. In order to evaluate most reliably the usefulness and discriminatory potential of typing methods, we selected potentially closely related strains (the analysis was limited to the local range), and the phenotypic resistance profile was used as a main criterion.

The selected MDR strains of *E*. *faecalis* were resistant to antimicrobials belonging to at least six different groups of antimicrobial agents. The phenotypic multidrug resistance profiles of *E*. *faecalis* were similar as those obtained from animals originated from the same herds, tested in previous study [24]. The resistance profile of all the strains comprised resistance to tetracycline, macrolides (including both erythromycin and tylosin), chloramphenicol, as well as high-level streptomycin and kanamycin. Such a pattern of phenotypic resistance has also been commonly observed among isolates from humans [33] and pigs, but in the case of animals with a much lower level of resistance to chloramphenicol [33,34].

Based on the varying degrees of phenotypic resistance to the other antimicrobials tested, three different phenotypic resistance profiles were determined and designated as A, B, and C. A majority of strains with a specific profile were associated with only one source of origin (the strains of profile A originated only from herd No1 and strains of profile B only from herd No 3). However, strains belonging to profile C were isolated from both herd No 2 and No 3, which initially may indicate that multidrug resistant strains may be transmitted not only between individuals within the same herd but also between different herds. This phenomenon seems to be confirmed by the high degree of correlation between the phenotypic resistance profile and the genomic profile of the strains tested, regardless of the source of origin. However, strains belonging to the same phenotypic profile were not fully genetically homogeneous, which may be due to diverse and multiple mechanisms of genetic resistance to the same antimicrobial.

For most of the analyzed antimicrobials (or for the antibiotics belonging to the same group), two or three different genes encoding resistance have been demonstrated. The tetracycline-resistant strains tested in this study carried *tet*M, but co-occurrence of both tetracycline resistance mechanisms (efflux encoded by *tet*L and ribosomal protection encoded by *tet*M) was observed in strains belonging to profile A and B. The co-existence of both types of resistance mechanisms may be reflected not only in the differentiated genotypic patterns (ADSRRS-fingerprinting patterns) but also as in our study, in higher MIC values of the strains [35,36]. The second most common type of resistance in *Enterococcus*, i.e. resistance to macrolides [37], was confirmed by the presence of the *erm*B gene in all the strains tested. However, the presence of another gene encoding target modification (*erm*F) was shown in four out of the nine strains belonging to profile A only. The *erm*F gene has been previously isolated in high percentage from *Enterococcus* strains originating from the living environment of pigs [38,39], which suggests that *erm*F may be common in isolates from this species of animals. Our studies also showed that all the strains were resistant to lincomycin, which was confirmed genetically by the presence of one or both *erm* genes (cross-resistance to macrolides, lincosamides, and streptogramin B) but another lincosamide-resistance mechanism encoded by the *lnu*B gene [40] was also found in all the strains of profile A and C. Although this gene seemed to be primarily characteristic for *E*. *faecium* only [41], the results of our study and those reported by other authors [42,43] confirmed its presence in a wider spectrum of *Enterococcus* species.

Although the level of phenotypic resistance to chloramphenicol was similar for all the strains tested (MIC ≥32μl ml^-1^), it was mediated by different genetic mechanisms. The resistance was mainly determined by *cat* genes encoding type A chloramphenicol acetyltransferases (strains belonging to profile A and C); however, in the case of six strains from profile C, two different cat genes (*cat*A-8 and *cat*A-9) were present simultaneously. The *cat*A genes are commonly found in gram-positive bacteria [44,45], but most authors have detected *cat*A genes mostly corresponding to the A-7 group in enterococci [33,45]. Nevertheless, the genes belonging to both *cat*A-9 and *cat*A-8 groups were detected in bacteria of the genus *Enterococcus* as well [45]. In turn, strains belonging to profile B had the *fex*A gene. This gene encoding different mechanisms of resistance mediates combined resistance to both phenicols: chloramphenicol and florfenicol [44].

Despite the wide panel of tested genes responsible for high-level aminoglycoside resistance, the genetic profile of this type of resistance was homogenous in all the strains. The resistance was encoded by the most typical genes: *aph(3’)-IIIa* (resistance to kanamycin), *ant(6)-Ia* (resistance to streptomycin), and *aac(6’)-Ie-aph(2”)-Ia* (resistance to all clinically available aminoglycosides but not streptomycin) [46]. These genes have been described as the most common mechanisms of HLAR in *Enterococcus* strains [46–48]. Other genes responsible for this type of resistance are rarely detected in *Enterococcus* [46,47] or are not widely evaluated [45,48].

The high percentage of concurrent prevalence of phenotypic resistance to macrolides, tetracyclines, and aminoglycosides in the strains tested in this study appears to be validated by the molecular analysis. Detection of the integrase gene in all the strains belonging to profile A and C may confirm the association between *tet*M, *erm*B, and *aph(3’)-IIIa* by Tn*1545*-like transposons [49,50]. This highly mobile conjugative transposon was reported previously among enterococci isolated mainly from humans and pigs [49]. However, this seems to be not the only mechanism allowing simultaneous spread of these genes. Werner et al. [51] described the presence of another gene cluster containing both *aph(3’)-IIIa* and *ant(6)-Ia* genes, disseminated in MDR *Enterococcus* strains. Moreover, Hidano et al.[52], who identified specific linkages between the presence of different genes encoding resistance to macrolides (*erm*B), tetracyclines (*tet*M, *tet*L), and aminoglycosides (*aph(3’)-IIIa* and *ant(6)-Ia)*, suggested that there may be varied genetic linkages between genes responsible for the multidrug resistance profile, and they are rather regular than random.

The profiles of virulence genes were less diversified (only three profiles) and strictly correlated with phenotypic resistance profiles. The linkage between resistance and presence of selected virulence determinants has been confirmed previously [53]. The genome of all the strains tested in our study included genes encoding sex pheromones (*cpd*, *cob*, *ccf*) and most of the strains (from profiles A and C) exhibited the presence of the *agg* gene. The *agg* gene was detected using the highly conserved sequence characteristic for representatives of the class of pheromone-responsive plasmids (*pAD1*, *pPD1*, and *pCF10*) [54,55]. The family of these plasmids shows high frequency of conjugative transfer of antibiotic resistance and virulence genes from donor cells by mating sex pheromones produced by potential recipient cells [53,54]. The aggregation substance promotes cell conjugation by bacterial aggregation, which results in close contact between the donor and recipient cells [56]. This phenomenon may increase virulence traits and antimicrobial resistance of recipient *E*. *faecalis* strains [53–55].

The use of only a method based on phenotypic resistance testing or only the presence of the resistance and virulence genes provided a low degree of differentiation within the pool of the tested strains (SID 0.386 and 0.783, respectively). According to the results of statistical analysis comparing only these two methods, it appears that, given the numerical rating indicating a high compatibility of the results obtained (60.3% and a total reproduction of the topography of the dendrogram of the resistance phenotype), it is sufficient to use the comparative analysis of gene profiles only (Wallace coefficient 1.0).

The molecular analysis performed with the ADSRRS-fingerprinting method revealed a high level of similarity within strains grouped in the particular resistance profiles, not exceeding the value of 0.93. Higher genomic variability, i.e. from 0,6 to 0,78, was noted between the specific profiles and reference strains (*E*. *faecalis* ATCC 29212 and *E*. *faecalis* ATCC 51299). Our study has shown that the ADSRRS-fingerprinting patterns are strongly determined by molecular resistance elements, which was also confirmed by previous analysis conducted for *E*. *faecalis* and *E*. *faecium* strains isolated from poultry [12]. Also for *E*. *faecalis*, *E*. *faecium* and *E*. *hirae* isolated from pigs [24] originated from the same herds as tested in present study, a similar correlation but between phenotypic resistance patterns and ADSRRS-fingerprinting profiles has been showed. However, comparing the genotypic profiles of multidrug resistant *E*. *faecalis* (ADSRRS-fingerprinting) from the current and previous study (samples for current analysis were collected about one year later than for previous analysis), the full homogeneity of ADSRRS profiles within the same phenotype of resistance has been shown in the previous study, while the strains in the present study were characterized by certain diversity within the same phenotype, both for ADSRRS profiles and resistance genes. This phenomenon may confirm the high discriminatory potential of ADSRRS-fingerprinting method allowing for diversification of strains with the same or similar phenotypic resistance profile, but differing in relation to the genes that determine this resistance [12]. A similar regularity in the consistency of genetic profiles (obtained with the PFGE method) with resistance profiles was observed by other authors [6,7,32,42,57]. This regularity seems to be especially important for analysis of strains with similar or the same antibiograms obtained from different sources (different farms, flocks, or individuals) and for tracking the ways of potential spread of multidrug resistant strains or strains causing local outbreaks. Also in this study, a significant genetic similarity between strains belonging to profile C but originating from two different sources (farms) has been found, probably related to highly similar profiles of resistance and virulence genes (difference in only one gene tested).

In this analysis, the ADSSRS- fingerprinting technique was applied due to its simplicity, ease, and speed of execution and, above all, high capacity of separation of closely related strains (SID 0.884). An unquestionable advantage of this method is the high Wallace coefficient in relation to the phenotypic and genotypic method (1.0 in both cases). Moreover, this method indicates a uniform way of grouping the isolates tested, confirmed by the Rand coefficient (50.2% and 89.8%, respectively), and thereby, it theoretically eliminates the need to use phenotypic resistance analysis and detection of genetic determinants at least for the pool of the multidrug resistant strains described in this study. On the other hand, phenotypic analysis of resistance cannot be omitted due to its crucial importance in the standard diagnostic procedure (it determines the potential success of therapy) and, as shown in our study, it is the first criterion for grouping and typing of multidrug resistant strains [27].

The gradual development of the proteomic technique MALDI-TOF MS has provided new opportunities for precise microbiological analysis. Numerous studies have shown that MALDI-TOF MS is a rapid, reliable, and cost-effective technique for identification of different groups of microorganisms [20,58–61], including *Enterococcus* [25,62,63]. In our study, we also obtained high log (score) values (> 2) for all the strains tested and the results were consistent with those of molecular identification.

A confirmation of the suitability of the MALDI-TOF MS technique in genus/species identification of microorganisms seems to be the determination of the genus-specific or species-specific biomarkers [64,65]. In our study, 25 different peaks were found in all the strains tested, including both reference strains. As in another study conducted by Quintela–Baluja et al. [64] and Santos et al. [65], a peak at m/z 4428 with high intensity visualized in pseudo-gel view was detected in all the strains, which confirms that this may be an important genus-specific marker [64]. Some other peaks detected by other authors exclusively in *E*. *faecalis* species (3036 m/z, 4764 m/z, 6077 m/z, 6857 m/z, and 9104 m/z) and those found in all the strains tested in this study may be considered as species-specific biomarkers [64,65]. The importance of other common peaks, especially the most intense ones, is currently unknown. Quintela–Baluja et al. [64] suggest that the expression of specific biomarkers may be related to ecological niches of strains tested. Giebel et al. [66] showed a correlation between the presence of specific peaks and species of animals from which a given *Enterococcus* strain originated. All our strains originated from the same species of animal and they were relatively similar within the phenotypic resistance profiles (with the exception of two reference strains); therefore, comprehensive analysis in this aspect needs a wider pool of strains tested, including different sources, species, and varied phenotypic and genotypic properties.

Attempts to use the MALDI-TOF MS method for typing strains below the species level undertaken during the past few years [17,67,68] often reached contradictory results, especially in the aspect of comparative analysis of antibiotic resistance mechanisms. Detection of specific antibiotic resistance mechanisms is questionable also in the case of analysis of *Enterococcus* resistance [9,17,19,69,70]. These discrepancies may be affected by several factors: differences between strains not relating to resistance only [18] (e.g. different profiles of virulence genes), different mechanisms of resistance to the same antimicrobials [17], and high molecular weight of some resistance factors, which may not be detectable in the peak range of standard analysis [23]. Moreover, the MALDI-TOF spectra include conserved molecules mostly corresponding to housekeeping and ribosomal proteins, which are only slightly modulated by environmental influences (e.g. selective pressure of antimicrobials). Therefore, clustering by this method does not always correspond to clustering by genetic methods [18] and does not always reproduce resistance profiles; however, due to the high discriminatory potential, it may complement other genetic techniques [71]. It seems that the strategy of the analysis of resistant strains with MALDI-TOF MS is most useful rather for rapid species identification of strains, allowing rapid assessment of the potential type of intrinsic antimicrobial resistance [20].

The highest resolving power was confirmed statistically also in our study by the highest Simpson coefficient of the discrimination of MALDI technique (0.941) among the analyzed methods along with the high compatibility of MALDI and ADSRRS-fingerprinting techniques, i.e. 84.4% (Rand coefficient 0.844), and a slight overlap of the groups in the MALDI technique (Wallace coefficient 0.082) versus ADSRRS-fingerprinting. However, the differences in Simpson's index of diversity between MALDI and ADSRRS-fingerprinting methods do not have statistical significance.

## Conclusion

The MALDI -TOF MS analysis showed higher discrimination power in diversification of multidrug resistant strains of *E*. *faecalis* (initially classified into three groups according to the resistance profile) than ADSRRS (0.941 vs. 0.884, respectively). However, the ADSRRS technique allowed reliable reproduction of the clustering pattern of isolates obtained with the technique of phenotypic analysis of resistance profiles and the analysis of resistance and virulence genes (Wallace coefficient 1.0). This feature seems to be the most applicable for epidemiological purposes and short-term analysis. Moreover, the agreement of the results from ADSRRS is at a higher level (50–90%) than in the case of MALDI (40–85%). This is probably related to the fact that both methods are based on completely different methodological bases. Since ADSRRS-fingerprinting is based on analyzing total genomic DNA diversity by detecting polymorphism restriction sites and MALDI -TOF MS spectra correspond rather to housekeeping proteins, it would be more appropriate to compare the results of the MALDI -TOF MS analysis with the results of multilocus sequence typing (MLST). This phenomenon has been confirmed by many authors who observed greater compatibility between MSP spectra and the type of ST [9,72–74] rather than the PFGE profile [21,75].

## Supporting Information

S1 Fig Dendrogram obtained after NTSYSpc analysis of phenotypic resistance profiles with the unweighted pair group method with arithmetic mean (UPGMA). (TIF)

S2 Fig Dendrogram obtained after NTSYSpc analysis of resistance and virulence gene profiles with the unweighted pair group method with arithmetic mean (UPGMA). (TIF)

S1 Table MIC values of multidrug resistant strains selected for this study. ^a^ AMP- ampicillin, CHL- chloramphenicol, CIP- ciprofloxacin, ENR- enrofloxacin, ERY–erythromycin, GEN- gentamicin, KAN- kanamycin, LIN- lincomycin, Q-D—quinupristin-dalfopristin, RIF- rifampin, STR- streptomycin, TET- tetracycline, TYL- tylosin, VAN- vancomycin.
^b^ The breakpoints (μl ml^-1^) for particular antimicrobials; for ampicillin, chloramphenicol, ciprofloxacin, erythromycin, gentamycin, rifampin, streptomycin, tetracycline and vancomycin the CLSI criteria (M100-S24) were used, for enrofloxacin, the breakpoint was defined according to VET 01-S2. Since CLSI does not define criteria for kanamycin, lincomycin and tylosin, the breakpoints defined by the National Antimicrobial Resistance Monitoring System Animal Isolates (NARMS) (http://www.ars.usda.gov/News/docs.htm?docid=6750&page=3) were used.
^c^ Profiles were created from the first letters of the names of antimicrobials (C -chloramphenicol, G-gentamicin, K-kanamycin,L- lincomycin, QD- quinupristin-dalfopristin, R-rifampin, S-streptomycin, T-tetracycline) or names of groups of antimicrobials: fluoroquinolones-F (ciprofloxacin, enrofloxacin) and macrolides-M (erythromycin, tylosin) to which given strains are resistant
(DOCX)

S2 Table Primers used in this study. (DOCX)

S3 Table The common peaks occured in all strains *E*. *faecalis* tested in this study. ^a^potential genus-specific marker
^b^potential species-specific markers.
(DOCX)

## References

[1] Werner G, Coque TM, Franz CM, Grohmann E, Hegstad K, Jensen L. et al Antibiotic resistant enterococci-tales of a drug resistance gene trafficker. Int J Med Microbiol. 2013; 303: 360–379 10.1016/j.ijmm.2013.03.001 23602510

[2] Aarestrup FM, Seyfarth AM, Emborg HD, Pedersen K, Hendriksen RS, Bager F. Effect of abolishment of the use of antimicrobial agents for growth promotion on occurrence of antimicrobial resistance in fecal enterococci from food animals in Denmark. Antimicrob Agents Chemother. 2001; 45: 2054–2059. 10.1128/AAC.45.7.2054-2059.2001 11408222 PMC90599

[3] Heuer OE, Hammerum AM, Collignon P, Wegener HC. Human health hazard from antimicrobial-resistant enterococci in animals and food. Clin Infect Dis. 2006; 43: 911–916. 10.1086/507534 16941376

[4] Hammerum AM. Enterococci of animal origin and their significance for public health. Clin Microbiol Infect. 2012; 18: 619–625 10.1111/j.1469-0691.2012.03829.x 22487203

[5] Tadesse DA, Zhao S, Tong E, Ayers S, Singh A, Bartholomew MJ. et al Antimicrobial drug resistance in *Escherichia coli* from humans and food animals, United States, 1950–2002. Emerg Infect Dis. 2012; 18: 741–749. 10.3201/eid1805.111153 22515968 PMC3358085

[6] Katsunuma Y, Hanazumi M, Fujisaki H, Minato H, Kataoka Y, Sawada T. et al Comparison of pulsed-field gel electrophoresis patterns of antimicrobial-resistant *Escherichia coli* and enterococci isolates from the feces of livestock and livestock farmers in Japan. J Gen Appl Microbiol. 2008; 54: 39–50. 18323680 10.2323/jgam.54.39

[7] Khan SA, Nawaz MS, Khan AA, Hopper SL, Jones RA, Cerniglia CE. Molecular characterization of multidrug-resistant *Enterococcus* spp. from poultry and dairy farms: Detection of virulence and vancomycin resistance gene markers by PCR. Mol Cell Probes. 2005; 19: 27–34. 10.1016/j.mcp.2004.09.001 15652217

[8] Krawczyk B, Lewandowski K, Bronk M, Samet A, Myjak P, Kur J. Evaluation of a novel method based on amplification of DNA fragments surrounding rare restriction sites (ADSRRS fingerprinting) for typing strains of vancomycin-resistant *Enterococcus faecium*. J Microbiol Meth. 2003; 52: 341–351. 10.1016/s0167-7012(02)00187-2 12531503

[9] Lasch P, Fleige C, Stämmler M, Layer F, Nübel U, Witte W. et al Insufficient discriminatory power of MALDI-TOF mass spectrometry for typing of *Enterococcus faecium* and *Staphylococcus aureus* isolates. J Microbiol Methods. 2014; 100: 58–69. 10.1016/j.mimet.2014.02.015 24614010

[10] Werner G. Molecular typing of enterococci/VRE. J Bacteriol Parasitol. 2013; 5

[11] Sabat AJ, Budimir A, Nashev D, Sá-Leão R, van Dijl Jm, Laurent F. et al Overview of molecular typing methods for outbreak detection and epidemiological surveillance. Euro Surveill. 2013; 24: 20380. 10.2807/ese.18.04.20380-en 23369389

[12] Nowakiewicz A, Ziółkowska G, Trościańczyk A, Zięba P, Gnat S. Determination of resistance and virulence genes in *Enterococcus faecalis* and *E*. *faecium* strains isolated from poultry and their genotypic characterization by ADSRRS-fingerprinting. Poultry Sci. 2016a; 00:1–11 10.3382/ps/pew365 27702915

[13] Krawczyk B, Leibner J, Barańska-Rybak W, Samet A, Nowicki R, Kur J. ADSRRS-fingerprinting and PCR MP techniques for studies of intraspecies genetic relatedness in *Staphylococcus aureus*. J Microbiol Meth. 2007; 71: 114–122. 10.1016/j.mimet.2007.08.010 17889385

[14] Krawczyk B, Naumiuk L, Lewandowski K, Baraniak A, Gniadkowski M, Samet A. et al Evaluation and comparison of random amplification of polymorphic DNA, pulsed-field gel electrophoresis and ADSRRS-fingerprinting for typing *Serratia marcescens* outbreaks. FEMS Immunol Med Microbiol. 2003; 38: 241–248. 14522459 10.1016/S0928-8244(03)00149-4

[15] Krutkiewicz A. Klimuszko D. Genotyping and PCR detection of potential virulence genes in *Campylobacter jejuni* and *Campylobacter coli* isolates from different sources in Poland. Folia Microbiol. 2010; 55: 167. 20490760 10.1007/s12223-010-0025-6

[16] Nowakiewicz A, Ziółkowska G, Zięba P, Gnat S, Wojtanowicz-Markiewicz K, Trościańczyk A. Coagulase-positive *Staphylococcus* isolated from wildlife: identification, molecular characterization and evaluation of resistance profiles with focus on a methicillin-resistant strain. Comp Immunol Microb. 2016; 44: 21–28. 10.1016/j.cimid.2015.11.003 26851590

[17] Kostrzewa M, Sparbier K, Maier T, Schubert S. MALDI-TOF MS: an upcoming tool for rapid detection of antibiotic resistance in microorganisms. Proteomics Clin Appl. 2013; 7: 767–778. 10.1002/prca.201300042 24123965

[18] Pulido MR, García-Quintanilla M, Martín-Peña R, Cisneros JM, McConnell MJ. Progress on the development of rapid methods for antimicrobial susceptibility testing. J Antimicrob Chemother. 2013; 68: 2710–2717. 10.1093/jac/dkt253 23818283

[19] Griffin PM, Price GR, Schooneveldt JM, Schlebusch S, Tilse MH, Urbanski T. et al Use of matrix-assisted laser desorption ionization-time of flight mass spectrometry to identify vancomycin-resistant enterococci and investigate the epidemiology of an outbreak. J Clin Microbiol. 2012; 50: 2918–2931. 10.1128/JCM.01000-12 22740710 PMC3421795

[20] Patel R. MALDI-TOF MS for the diagnosis of infectious diseases. Clin Chem. 2015; 61: 100–111. 10.1373/clinchem.2014.221770 25278500

[21] Rim JH, Lee Y, Hong SK, Park Y, Kim M, D'Souza R. et al Insufficient discriminatory power of matrix-assisted laser desorption ionization time-of-flight mass spectrometry dendrograms to determine the clonality of multi-drug-resistant *Acinetobacter baumannii* isolates from an intensive care unit. Biomed Res Int. 2015; 2015: 535027 10.1155/2015/535027 26101775 PMC4458526

[22] Singhal N, Kumar M, Kanaujia PK, Virdi JS. MALDI-TOF mass spectrometry: an emerging technology for microbial identification and diagnosis. Front Microbiol. 2015; 6:791 10.3389/fmicb.2015.00791 26300860 PMC4525378

[23] Spinali S, van Belkum A, Goering RV, Girard V, Welker M, Van Nuenen M. et al Microbial typing by matrix-assisted laser desorption ionization-time of flight mass spectrometry: do we need guidance for data interpretation? J Clin Microbiol. 2015; 53: 760–765. 10.1128/JCM.01635-14 25056329 PMC4390642

[24] Nowakiewicz A, Ziółkowska G, Trościańczyk A, Zięba P, Gnat S. Determination of antimicrobial resistance of *Enterococcus* strains isolated from pigs and their genotypic characterization by method of amplification of DNA fragments surrounding rare restriction sites (the ADSRRS-fingerprinting). J Med Microbiol. 2016; 10.1099/jmm.0.000400 28260584

[25] Nowakiewicz A, Ziółkowska G, Zięba P, Trościańczyk A, Banach T, Kowalski C. Modified 16S–23S rRNA intergenic region restriction endonuclease analysis for species identification of *Enterococcus* strains isolated from pigs, compared with identification using classical methods and matrix-assisted laser desorption/ionization time-of-flight mass spectrometry. J Med Microbiol. 2015; 64: 217–223. 10.1099/jmm.0.000008 25587074

[26] Clinical and Laboratory Standard Institute. M100- S24. Performance Standards for Antimicrobial Susceptibility Testing; Twenty fourth Informational Supplement. 2014; 34(1).

[27] Magiorakos A-P, Srinivasan A, Carey RB, Carmeli Y, Falagas ME, Giske CG. et al Multidrug-resistant, extensively drug-resistant and pandrug-resistant bacteria: an international expert proposal for interim standard definitions for acquired resistance. Clin Microbiol Infect. 2012; 18: 268–281. 10.1111/j.1469-0691.2011.03570.x 21793988

[28] Novais C, Freitas AR, Silveira E, Antunes P, Silva R, Coque TM. et al Spread of multidrug-resistant *Enterococcus* to animals and humans:an underestimated role for the pig farm environment. J Antimicrob Chemother. 2013; 68: 2746–2754 10.1093/jac/dkt289 23861310

[29] Hummel A, Holzapfel WH, Franz CMAP. Characterization and transfer of antibiotic resistance genes from enterococci isolated from food Syst Appl Microbiol. 2007; 30: 1–7 10.1016/j.syapm.2006.02.004 16563685

[30] Miller WR, Munita JM, Arias CA. Mechanisms of antibiotic resistance in enterococci Expert Rev Anti Infect Ther. 2014; 12: 1221–1236. 10.1586/14787210.2014.956092 25199988 PMC4433168

[31] Guzman Prieto AM, van Schaik W, Rogers MRC, Coque TM, Baquero F, Corander J. et al Global emergence and dissemination of enterococci as nosocomial pathogens: attack of the clones? Front Microbiol. 2016; 7:788 10.3389/fmicb.2016.00788 27303380 PMC4880559

[32] Dicuonzo G, Gherardi G, Lorino G, Angeletti S, Battistoni F, Bertuccini L. et al Antibiotic resistance and genotypic characterization by PFGE of clinical and environmental isolates of enterococci. FEMS Microbiol Lett. 2001; 201: 205–211. 11470363 10.1111/j.1574-6968.2001.tb10758.x

[33] Aarestrup FM, Agerso Y, Gerner-Smidt P, Madsen M, Jensen LB. Comparison of antimicrobial resistance phenotypes and resistance genes in *Enterococcus faecalis* and *Enterococcus faecium* from humans in the community, broilers and pigs in Denmark. Diagn Microbiol Infect Dis. 2000; 37: 127–137. 10863107 10.1016/s0732-8893(00)00130-9

[34] Tremblay CL, Letellier A, Quessy S, Daignault D, Archambault M. Antibiotic-resistant *Enterococcus faecalis* in abattoir pigs and plasmid colocalization and cotransfer of *tet*(M) and *erm*(B) genes. J Food Prot. 2012; 75: 1595–602. 10.4315/0362-028X.JFP-12-047 22947466

[35] Huys G, D'Haene K, Collard JM, Swings J. Prevalence and molecular characterization of tetracycline resistance in *Enterococcus* isolates from food. Appl Environ Microbiol. 2004; 70: 1555–1562. 10.1128/AEM.70.3.1555-1562.2004 15006778 PMC368340

[36] Schwaiger K, Harms K, Hölzel C, Meyer K, Karl M, Bauer J. Tetracycline in liquid manure selects for co-occurrence of the resistance genes *tet*(M) and *tet*(L) in *Enterococcus faecalis*. Vet Microbiol. 2009; 18: 386–392. 10.1016/j.vetmic.2009.06.005 19570622

[37] Fard RMN, Heuzenroeder MW, Barton MD. Antimicrobial and heavy metal resistance in commensal enterococci isolated from pigs Vet Microbiol. 2011; 148: 276–282 10.1016/j.vetmic.2010.09.002 20951513

[38] Hoang TTT, Soupir ML, Liu P, Bhandari A. Occurrence of tylosin-resistant enterococci in swine manure and tile drainage systems under no-till management Water Air Soil Pollut. 2013; 224: 1754.

[39] Sapkota AR, Ojo KK, Roberts MC, Schwab KJ. Antibiotic resistance genes in multidrug-resistant *Enterococcus* spp. and *Streptococcus* spp. recovered from the indoor air of a large-scale swine-feeding operation. Lett Appl Microbiol. 2006; 43: 534–540. 10.1111/j.1472-765X.2006.01996.x 17032228

[40] Garrido A. Galvez MA, Pulido RP. Antimicrobial resistance in enterococci. J Infect Dis Ther. 2014; 2: 150.

[41] Bozdogan B, Berrezouga L, Kuo MS, Yurek DA, Farley KA, Stockman BJ. et al A new resistance gene, *lin*B, conferring resistance to lincosamides by nucleotidylation in *Enterococcus faecium* HM1025. Antimicrob Agents Chemother. 1999; 43: 925–929. 10103201 10.1128/aac.43.4.925 PMC89227

[42] Jackson CR, Fedorka-Cray PJ, Barrett JB, Brousse JH, Gustafson J, Kucher M. Mechanisms of antimicrobial resistance and genetic relatedness among enterococci isolated from dogs and cats in the United States. J Appl Microbiol. 2010; 108: 2171–2179. 10.1111/j.1365-2672.2009.04619.x 19968729

[43] Tremblay C-L, Letellier A, Quessy S, Boulianne M, Daignault D, Archambault M. Multiple-antibiotic resistance of *Enterococcus faecalis* and *Enterococcus faecium* from cecal contents in broiler chicken and turkey flocks slaughtered in Canada and plasmid colocalization of *tet*O and *erm*B genes. J Food Protect. 2011; 74: 1639–1648. 10.4315/0362-028X.JFP-10-451 22004810

[44] Schwarz S, Kehrenberg C, Doublet B, Cloeckaert A. Molecular basis of bacterial resistance to chloramphenicol and florfenicol. FEMS Microbiol Rev. 2004; 28: 519–42. 10.1016/j.femsre.2004.04.001 15539072

[45] Seputiene V, Bogdaite A, Ruzauskas M, Suziedeliene E. Antibiotic resistance genes and virulence factors in *Enterococcus faecium* and *Enterococcus faecalis* from diseased farm animals: pigs, cattle and poultry. Pol J Vet Sci. 2012; 15: 431–438. 23214361

[46] Chow JW. Aminoglycoside resistance in enterococci. Clin Infect Dis. 2000; 31: 586–589 10.1086/313949 10987725

[47] Jackson CR, Fedorka-Cray PJ, Barrett JB, Ladely SR. High-level aminoglycoside resistant enterococci isolated from swine. Epidemiol Infect. 2005; 133: 367–371. 15816164 10.1017/s0950268804003395 PMC2870258

[48] Ramos S, Igrejas G, Capelo-Martinez J, Poeta P. Antibiotic resistance and mechanisms implicated in fecal enterococci recovered from pigs, cattle and sheep in a Portuguese slaughterhouse. Ann Microbiol. 2012; 62: 1485.

[49] De Leener E, Martel A, Decostere A, Haesebrouck F. Distribution of the *erm* (B) gene, tetracycline resistance genes, and *Tn1545*-like transposons in macrolide- and lincosamide-resistant enterococci from pigs and humans. Microb Drug Resist. 2004; 10: 341–345. 10.1089/mdr.2004.10.341 15650380

[50] Rice LB. Tn916 family conjugative transposons and dissemination of antimicrobial resistance determinants. Antimicrob Agents Chemother. 1998; 42: 1871–1877. 9687377 10.1128/aac.42.8.1871 PMC105703

[51] Werner G, Hildebrandt B, Witte W. Aminoglycoside-streptothricin resistance gene cluster *aad*E-*sat*4-*aph*A-3 disseminated among multiresistant isolates of *Enterococcus faecium*. Antimicrob Agents Chemother. 2001;45: 3267–3269. 10.1128/AAC.45.11.3267-3269.2001 11600397 PMC90823

[52] Hidano A, Yamamoto T, Hayama Y, Muroga N, Kobayashi S, Nishida T. et al Unraveling antimicrobial resistance genes and phenotype patterns among *Enterococcus faecalis* isolated from retail chicken products in Japan. PLOS ONE. 2015; 10(3): e0121189 10.1371/journal.pone.0121189 25781022 PMC4363150

[53] Choi JM, Woo GJ. Transfer of tetracycline resistance genes with aggregation substance in food-borne *Enterococcus faecalis*. Curr Microbiol. 2015;70: 476–484. 10.1007/s00284-014-0742-1 25487115 PMC4338359

[54] Chandler JR, Dunny GM. Enterococcal peptide sex pheromones: synthesis and control of biological activity. Peptides. 2004; 25:1377–1388. 10.1016/j.peptides.2003.10.020 15374642

[55] Eaton TJ, Gasson MJ. Molecular screening of *Enterococcus* virulence determinants and potential for genetic exchange between food and medical isolates. Appl Environ Microbiol. 2001; 67: 1628–1635. 10.1128/AEM.67.4.1628-1635.2001 11282615 PMC92779

[56] Sava IG, Heikens E, Huebner J. Pathogenesis and immunity in enterococcal infections. Clin Microbiol Infect. 2010; 16: 533–540. 10.1111/j.1469-0691.2010.03213.x 20569264

[57] Corso AC, Gagetti PS, Rodrıguez MM, Melano RG, Ceriana PG, Faccone DF. et al Molecular epidemiology of vancomycin-resistant *Enterococcus faecium* in Argentina. Int J Infect Dis. 2007; 11: 69–75 10.1016/j.ijid.2006.02.003 16793306

[58] Adaszek Ł, Banach T, Bartnicki M, Winiarczyk D, Łyp P, Winiarczyk S. Application the mass spectrometry MALDI-TOF technique for detection of *Babesia canis* infection in dogs. Parasitol Res. 2014; 113: 4293–4295 10.1007/s00436-014-4124-1 25238794 PMC4200353

[59] Becker PT, de Bel A, Martiny D, Ranque S, Piarroux R, Cassagne C. et al Identification of filamentous fungi isolates by MALDI-TOF mass spectrometry: clinical evaluation of an extended reference spectra library. Med Mycol. 2014; 52: 826–834. 10.1093/mmy/myu064 25349253

[60] Emonet S, Shah HN, Cherkaoui A, Schrenzel J. Application and use of various mass spectrometry methods in clinical microbiology. Clin Microbiol Infect 2010; 16: 1604–1613 10.1111/j.1469-0691.2010.03368.x 20969670

[61] da Silva Paim TG, Reiter KC, de Oliveira CF, d’Azevedo PA. MALDI-TOF MS performance to identify gram-positive cocci clinical isolates in Porto Alegre. Brazil J Infect Control. 2013; 2: 112–116.

[62] Werner G, Fleige C, Fessler AT, Timke M, Kostrzewa M, Zischka M. et al Improved identification including MALDI-TOF mass spectrometry analysis of group D streptococci from bovine mastitis and subsequent molecular characterization of corresponding *Enterococcus faecalis* and *Enterococcus faecium* isolates. Vet Microbiol. 2012; 9:160–162 10.1016/j.vetmic.2012.05.019 22677481

[63] Stępień-Pyśniak D, Banach T, Adaszek Ł, Pyzik E, Wilczyński J. et al Prevalence and antibiotic resistance of *Enterococcus* strains isolated from poultry. Acta Vet Hung. 2016; 64: 148–163. 10.1556/004.2016.016 27342087

[64] Quintela-Baluja M. Bohme K, Fernandez-No IC, Morandi S, Alnakip ME, Caamano-Antelo S. et al Characterization of different food-isolated *Enterococcus* strains by MALDI-TOF mass fingerprinting. Electrophoresis 2013; 34: 2240–2250 10.1002/elps.201200699 23712773

[65] Santos T, Capelo JL, Santos HM, Oliveira I, Marinho C, Gonçalves A. et al Use of MALDI-TOF mass spectrometry fingerprinting to characterize *Enterococcus* spp. and *Escherichia coli* isolates. J Proteomics. 2015; 8:321–331. 10.1016/j.jprot.2015.02.017 25753124

[66] Giebel RA, Fredenberg W, Sandrin TR. Characterization of environmental isolates of *Enterococcus* spp. by matrix-assisted laser desorption/ionization time-of-flight mass spectrometry. Water Res. 2008; 42: 931–940. 10.1016/j.watres.2007.09.005 17931682

[67] Sandrin TR, Goldstein JE, Schumaker S. MALDI TOF MS profiling of bacteria at the strain level: a review. Mass Spectrom Rev. 2012; 10.1002/mas.21359 22996584

[68] Murray PR. Matrix-assisted laser desorption ionization time-of-flight mass spectrometry: usefulness for taxonomy and epidemiology. Clin Microbiol Infect. 2010; 16: 1626–1630. 10.1111/j.1469-0691.2010.03364.x 20825435

[69] Nakano S, Matsumura Y, Kato K, Yunoki T, Hotta G, Noguchi T. et al Differentiation of vanA-positive *Enterococcus faecium* from *van*A-negative *E*. *faecium* by matrix-assisted laser desorption/ionisation time-of-flight mass spectrometry.Int J Antimicrob Agents. 2014; 44: 256–259. 10.1016/j.ijantimicag.2014.05.006 25104134

[70] Mutters NT, Burckhardt I, Heeg. MALDI-TOF Mass Spectrometry as a tool for epidemiological outbreak analysis–Can it work?. J Med Diagn Meth. 2015; 4: 1000.184.

[71] Hrabák J, Chudácková E, Walková R. Matrix-assisted laser desorption ionization-time of flight (MALDI-TOF) mass spectrometry for detection of antibiotic resistance mechanisms: from research to routine diagnosis. Clin Microbiol Rev. 2013; 26: 103–114. 10.1128/CMR.00058-12 23297261 PMC3553667

[72] Josten M, Reif M, Szekat C, Al-Sabti N, Roemer T, Sparbier K. et al Analysis of the matrix-assisted laser desorption ionization-time of flight mass spectrum of *Staphylococcus aureus* identifies mutations that allow differentiation of the main clonal lineages. J Clin Microbiol. 2013; 51: 1809–1817. 10.1128/JCM.00518-13 23554199 PMC3716067

[73] Shin HB, Yoon J, Lee Y, Kim MS, Lee K. Comparison of MALDI-TOF MS, housekeeping gene sequencing, and 16S rRNA gene sequencing for identification of *Aeromonas* clinical isolates. Yonsei Med J. 2015; 56: 550–555. 10.3349/ymj.2015.56.2.550 25684008 PMC4329371

[74] Nagy E, Urbán E, Becker S, Kostrzewa M, Vörös A, Hunyadkürti J. et al MALDI-TOF MS fingerprinting facilitates rapid discrimination of phylotypes I, II and III of *Propionibacterium acnes*. Anaerobe 2013; 20: 20e26 23485355 10.1016/j.anaerobe.2013.01.007

[75] Novais A, Sousa C, de Dios Caballero J, Fernandez-Olmos A, Lopes J, Ramos H. et al MALDI-TOF mass spectrometry as a tool for the discrimination of high-risk *Escherichia coli* clones from phylogenetic groups B2 (ST131) and D (ST69, ST405, ST393). Eur J Clin Microbiol Infect Dis. 2014; 33: 1391–1399. 10.1007/s10096-014-2071-5 24599708

