# Supplementary figures and images for: Characterization of Multidrug Resistant E. faecalis Strains from Pigs of Local Origin by ADSRRS-Fingerprinting and MALDI -TOF MS; Evaluation of the Compatibility of Methods Employed for Multidrug Resistance Analysis
Source: PLoS One. 2017 Jan 30;12(1):e0171160. doi: 10.1371/journal.pone.0171160 (PMC5279778; doi:10.1371/journal.pone.0171160)

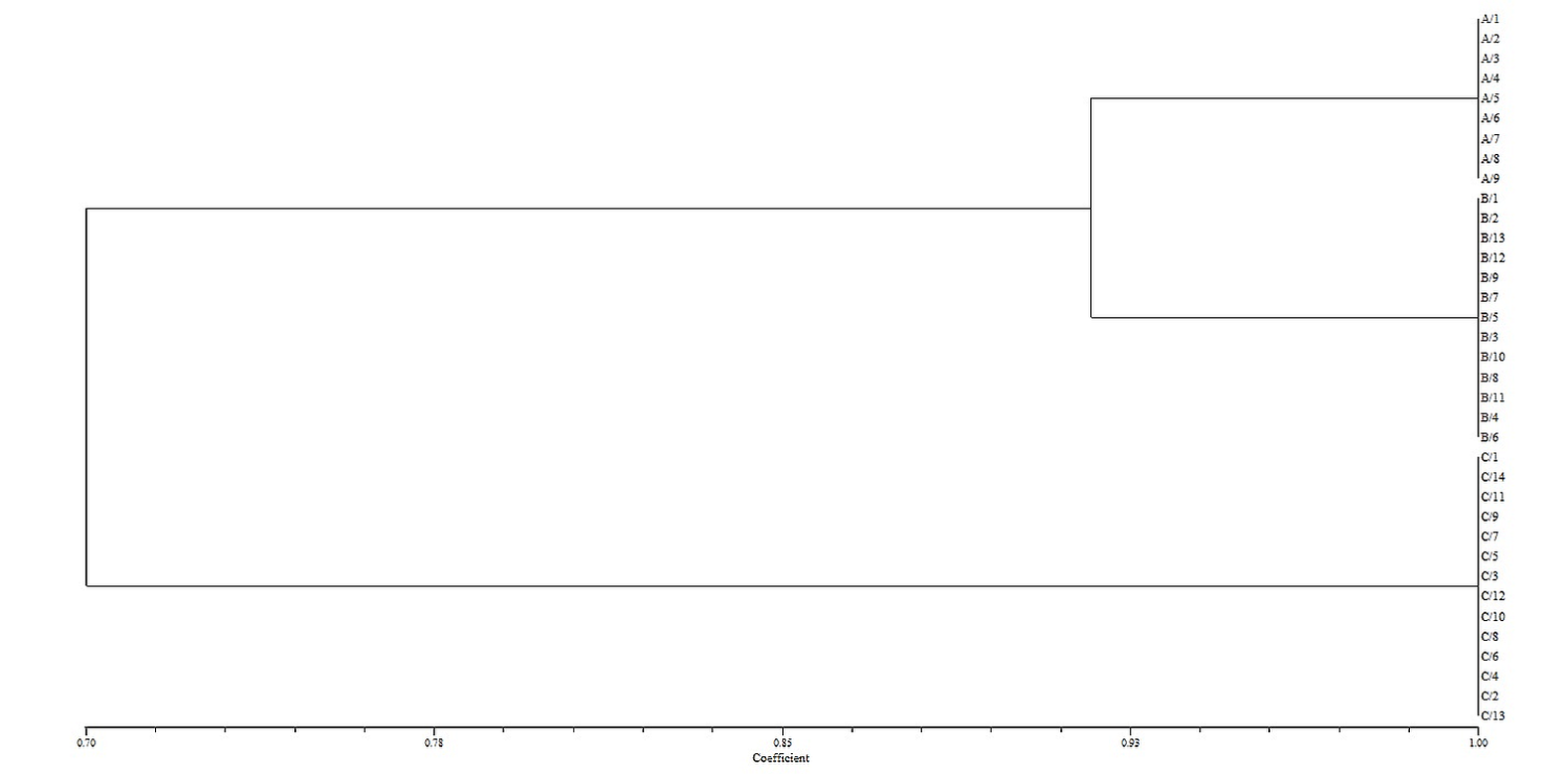

Supplement: S1 Fig — (TIF) [file pone.0171160.s001.tif]

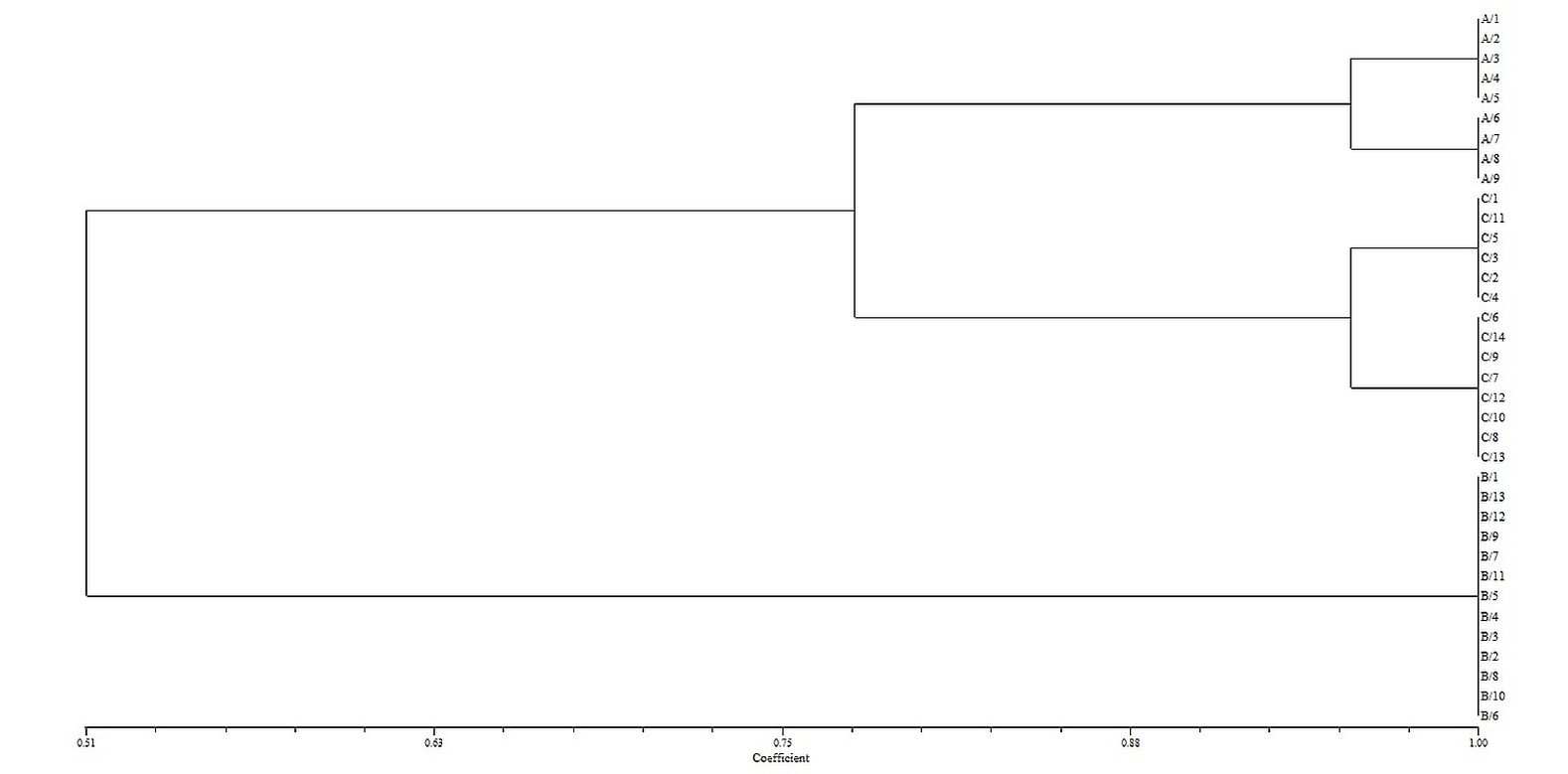

Supplement: S2 Fig — (TIF) [file pone.0171160.s002.tif]
