# Supplementary material for: Characterization of Multidrug Resistant E. faecalis Strains from Pigs of Local Origin by ADSRRS-Fingerprinting and MALDI -TOF MS; Evaluation of the Compatibility of Methods Employed for Multidrug Resistance Analysis
Source: PLoS One. 2017 Jan 30;12(1):e0171160. doi: 10.1371/journal.pone.0171160 (PMC5279778; doi:10.1371/journal.pone.0171160)
Supplement: S1 Table — a AMP- ampicillin, CHL- chloramphenicol, CIP- ciprofloxacin, ENR- enrofloxacin, ERY–erythromycin, GEN- gentamicin, KAN- kanamycin, LIN- lincomycin, Q-D—quinupristin-dalfopristin, RIF- rifampin, STR- streptomycin, TET- tetracycline, TYL- tylosin, VAN- vancomycin. b The breakpoints (μl ml-1) for particular antimicrobials; for ampicillin, chloramphenicol, ciprofloxacin, erythromycin, gentamycin, rifampin, streptomycin, tetracycline and vancomycin the CLSI criteria (M100-S24) were used, for enrofloxacin, the breakpoint was defined according to VET 01-S2. Since CLSI does not define criteria for kanamycin, lincomycin and tylosin, the breakpoints defined by the National Antimicrobial Resistance Monitoring System Animal Isolates (NARMS) (http://www.ars.usda.gov/News/docs.htm?docid=6750&page=3) were used. c Profiles were created from the first letters of the names of antimicrobials (C -chloramphenicol, G-gentamicin, K-kanamycin,L- lincomycin, QD- quinupristin-dalfopristin, R-rifampin, S-streptomycin, T-tetracycline) or names of groups of antimicrobials: fluoroquinolones-F (ciprofloxacin, enrofloxacin) and macrolides-M (erythromycin, tylosin) to which given strains are resistant (DOCX) [file pone.0171160.s003.docx]

| Lp | Strain | Source(No of farm) | AMP^a^≥ 16^b^ | CHL ≥32 | CIP ≥ 4 | ENR≥ 4 | ERY≥ 8 | GEN ≥ 512 | KAN ≥ 1024 | LIN ≥ 8 | Q-D ≥ 4 | RIF ≥ 4 | STR ≥ 1024 | TET ≥ 16 | TYL ≥ 32 | VAN≥ 32 | Pattern of phenotypic resistance^c^ |
| --- | --- | --- | --- | --- | --- | --- | --- | --- | --- | --- | --- | --- | --- | --- | --- | --- | --- |
| 1 | A/1 | 1 | 0,5 | >32 | 32 | 32 | >128 | >2048 | >2048 | 16 | 8 | 1 | >2048 | 128 | >128 | 2 | CFMGKLQDST |
| 2 | A/2 | 1 | 0,25 | >32 | 32 | 32 | >128 | >2048 | >2048 | 16 | 8 | 1 | >2048 | 128 | >128 | 2 | CFMGKLQDST |
| 3 | A/3 | 1 | 0,5 | >32 | 32 | 32 | >128 | >2048 | >2048 | 32 | 8 | 1 | >2048 | 128 | >128 | 2 | CFMGKLQDST |
| 4 | A/4 | 1 | 0,25 | >32 | 32 | 32 | >128 | >2048 | >2048 | 16 | 8 | 1 | >2048 | 128 | >128 | 2 | CFMGKLQDST |
| 5 | A/5 | 1 | 0,125 | >32 | 32 | 32 | >128 | >2048 | >2048 | 32 | 4 | 1 | >2048 | 128 | >128 | 1 | CFMGKLQDST |
| 6 | A/6 | 1 | 0,5 | >32 | 32 | 16 | >128 | 1024 | >2048 | 32 | 4 | 1 | >2048 | 128 | >128 | 1 | CFMGKLQDST |
| 7 | A/7 | 1 | 0,25 | >32 | 32 | 32 | >128 | >2048 | >2048 | 32 | 8 | 1 | >2048 | 128 | >128 | 1 | CFMGKLQDST |
| 8 | A/8 | 1 | 0,5 | >32 | 32 | 32 | >128 | >2048 | >2048 | 32 | 8 | 1 | >2048 | 128 | >128 | 1 | CFMGKLQDST |
| 9 | A/9 | 1 | 0,5 | >32 | 16 | 32 | >128 | >2048 | >2048 | 32 | 8 | 1 | >2048 | 64 | >128 | 1 | CFMGKLQDST |
| 10 | B/1 | 3 | 0,5 | >32 | 32 | 32 | >128 | 64 | >2048 | 16 | 4 | 1 | >2048 | >128 | >128 | 2 | CFMKLQDST |
| 11 | B/2 | 3 | 0,125 | >32 | 32 | 32 | >128 | 16 | >2048 | 32 | 4 | 0,125 | >2048 | >128 | >128 | 0,5 | CFMKLQDST |
| 12 | B/3 | 3 | 0,5 | >32 | 32 | 32 | >128 | 64 | >2048 | 32 | 4 | 1 | >2048 | >128 | >128 | 2 | CFMKLQDST |
| 13 | B/4 | 3 | 0,25 | >32 | 16 | 16 | >128 | 32 | >2048 | 32 | 4 | 1 | >2048 | 128 | >128 | 2 | CFMKLQDST |
| 14 | B/5 | 3 | 0,5 | >32 | >32 | 32 | >128 | 32 | >2048 | 16 | 4 | 1 | >2048 | >128 | >128 | 2 | CFMKLQDST |
| 15 | B/6 | 3 | 1 | 32 | 32 | 32 | >128 | 32 | >2048 | 32 | 4 | 1 | >2048 | 128 | >128 | 2 | CFMKLQDST |
| 16 | B/7 | 3 | 1 | >32 | 32 | 32 | >128 | 32 | >2048 | 32 | 4 | 1 | >2048 | 128 | >128 | 2 | CFMKLQDST |
| 17 | B/8 | 3 | 0,5 | 32 | 32 | 32 | >128 | 32 | >2048 | 32 | 4 | 1 | >2048 | 128 | >128 | 2 | CFMKLQDST |
| 18 | B/9 | 3 | 1 | 32 | 32 | 32 | >128 | 32 | >2048 | 32 | 4 | 1 | >2048 | 128 | >128 | 2 | CFMKLQDST |
| 19 | B/10 | 3 | 1 | 32 | 32 | 32 | >128 | 64 | >2048 | 32 | 8 | 1 | >2048 | >128 | >128 | 2 | CFMKLQDST |
| 20 | B/11 | 3 | 1 | 32 | 32 | 32 | >128 | 64 | >2048 | 32 | 4 | 1 | >2048 | >128 | >128 | 2 | CFMKLQDST |
| 21 | B/12 | 3 | 1 | 32 | 32 | 32 | >128 | 64 | >2048 | 32 | 4 | 1 | >2048 | >128 | >128 | 2 | CFMKLQDST |
| 22 | B/13 | 3 | 1 | 32 | 32 | 32 | >128 | 64 | >2048 | 32 | 4 | 1 | >2048 | >128 | >128 | 2 | CFMKLQDST |
| 23 | C/1 | 2 | 1 | 32 | 0,125 | 0,5 | >128 | 2048 | >2048 | 16 | 4 | 8 | >2048 | 64 | >128 | 1 | CMGKLQDRST |
| 24 | C/2 | 2 | 1 | 32 | 0,25 | 0,25 | >128 | 2048 | >2048 | 16 | 8 | 8 | >2048 | 64 | >128 | 1 | CMGKLQDRST |
| 25 | C/3 | 2 | 1 | 32 | 0,25 | 0,5 | >128 | 2048 | >2048 | 32 | 4 | 8 | >2048 | 64 | >128 | 1 | CMGKLQDRST |
| 26 | C/4 | 2 | 1 | >32 | 0,5 | 0,125 | >128 | 2048 | >2048 | 16 | 4 | 8 | >2048 | 64 | >128 | 1 | CMGKLQDRST |
| 27 | C/5 | 2 | 1 | 32 | 0,5 | 0,25 | >128 | 2048 | >2048 | 16 | 4 | 8 | >2048 | 64 | >128 | 1 | CMGKLQDRST |
| 28 | C/6 | 3 | 1 | >32 | 0,125 | 0,5 | >128 | 2048 | >2048 | 16 | 8 | 8 | >2048 | 32 | >128 | 1 | CMGKLQDRST |
| 29 | C/7 | 3 | 1 | >32 | 0,5 | 0,125 | >128 | 2048 | >2048 | 16 | 4 | 8 | >2048 | 32 | >128 | 1 | CMGKLQDRST |
| 30 | C/8 | 3 | 1 | >32 | 0,5 | 0,5 | >128 | 2048 | >2048 | 32 | 4 | 8 | >2048 | 64 | >128 | 1 | CMGKLQDRST |
| 31 | C/9 | 3 | 1 | >32 | 0,25 | 0,5 | >128 | 2048 | >2048 | 16 | 8 | 8 | >2048 | 32 | >128 | 1 | CMGKLQDRST |
| 32 | C/10 | 3 | 1 | >32 | 0,5 | 0,25 | >128 | 2048 | >2048 | 16 | 4 | 8 | >2048 | 32 | >128 | 1 | CMGKLQDRST |
| 33 | C/11 | 2 | 1 | >32 | 0,25 | 0,25 | >128 | 2048 | >2048 | 32 | 8 | 16 | >2048 | 64 | >128 | 1 | CMGKLQDRST |
| 34 | C/12 | 3 | 0,5 | >32 | 1 | 0,5 | >128 | 2048 | >2048 | 16 | 4 | 8 | >2048 | 32 | >128 | 1 | CMGKLQDRST |
| 35 | C/13 | 3 | 0,5 | >32 | 1 | 0,5 | >128 | 2048 | >2048 | 16 | 4 | 8 | >2048 | 32 | >128 | 2 | CMGKLQDRST |
| 36 | C/14 | 3 | 1 | >32 | 0,25 | 0,25 | >128 | 2048 | >2048 | 16 | 4 | 8 | >2048 | 32 | >128 | 1 | CMGKLQDRST |

S1 Table. MIC values of multidrug resistant strains selected for this study

^a^ AMP- ampicillin, CHL- chloramphenicol, CIP- ciprofloxacin, ENR- enrofloxacin, ERY – erythromycin, GEN- gentamicin, KAN- kanamycin, LIN- lincomycin, Q-D - quinupristin-dalfopristin, RIF- rifampin, STR- streptomycin, TET- tetracycline, TYL- tylosin, VAN- vancomycin.

^b^ The breakpoints (µl ml^-1^) for particular antimicrobials; for ampicillin , chloramphenicol , ciprofloxacin, erythromycin, gentamycin , rifampin, streptomycin, tetracycline and vancomycin the CLSI criteria (M100-S24) were used, for enrofloxacin, the breakpoint was defined according to VET 01-S2. Since CLSI does not define criteria for kanamycin, lincomycin and tylosin, the breakpoints defined by the National Antimicrobial Resistance Monitoring System Animal Isolates (NARMS) (<http://www.ars.usda.gov/News/docs.htm?docid=6750&page=3>) were used.

^c^ Profiles were created from the first letters of the names of antimicrobials (C -chloramphenicol, G-gentamicin, K-kanamycin,L- lincomycin, QD- quinupristin-dalfopristin ,R-rifampin, S-streptomycin, T-tetracycline) or names of groups of antimicrobials: fluoroquinolones-F (ciprofloxacin, enrofloxacin) and macrolides-M (erythromycin, tylosin) to which given strains are resistant
