# Supplementary material for: Characterization of Multidrug Resistant E. faecalis Strains from Pigs of Local Origin by ADSRRS-Fingerprinting and MALDI -TOF MS; Evaluation of the Compatibility of Methods Employed for Multidrug Resistance Analysis
Source: PLoS One. 2017 Jan 30;12(1):e0171160. doi: 10.1371/journal.pone.0171160 (PMC5279778; doi:10.1371/journal.pone.0171160)
Supplement: S2 Table — (DOCX) [file pone.0171160.s004.docx]

S1 Table Primers used in this study

| Gene detected | Sequences of the primer (5’ to 3’) | Amplicon size (bp) | Reference |
| --- | --- | --- | --- |
| *tnd*X (Tn*5397)* | F: ATGATGGGTTGGACAAAGA R: CTTTGCTCGATAGGCTCTA | 610 | Agersø et al., 2006 |
| *Int-Tn* (Tn*916/*Tn*1545)* | F: GCGTGATTGTATCTCACT R: GACGCTCCTGTTGCTTCT | 1,028 | Doherty et al., 2000 |
| *aac(6’)-Ie-aph(2”)-Ia* | F: CCAAGAGCAATAAGGGCATA R: CACTATCATACCACTACCG | 220 | Van de Klundert and Vliegenthart, 1993 |
| aph(2")-Ib | F: CTTGGACGCTGAGATATATGAGCAC R: GTTTGTAGCAATTCAGAAACACCCTT | 867 | Vakulenko et al. 2003 |
| aph(2")-Ic | F: CCACAATGATAATGACTCAGTTCCC R: CCACAGCTTCCGATAGCAAGAG | 444 | Vakulenko et al. 2003 |
| aph(2")-Id | F: GTGGTTTTTACAGGAATGCCATC R: CCCTCTTCATACCAATCCATATAACC | 641 | Vakulenko et al. 2003 |
| *aph(3’)-IIIa* | F: GCCGATGTGGATTGCGAAAA R: GCTTGATCCCCAGTAAGTCA | 292 | Van de Klundert and Vliegenthart, 1993 |
| *aad*A | F: TGATTTGCTGGTTACGGTGAC R: CGCTATGTTCTCTTGCTTTTG | 284 | Clark et al., 1999 |
| *ant(6)-Ia* | F: ACTGGCTTAATCAATTTGGG R: GCCTTTCCGCCACCTCACCG | 577 | Del Campo et al., 2000 |
| *ant*(*4*’)-*Ia* | F: GGAAGCAGAGTTCAGCCATG R: TGCCTGCATATTCAAACAGC | 266 | Matsumura et al. 1984 |
| *ant*(*9*)-*Ia* | F: GGTTCAGCAGTAAATGGTGGT R: TGCCACATTCGAGCTAGGGTT | 476 | Murphy 1985 |
| *erm*(A) | F:TCTAAAAAGCATGTAAAAGAA R: CTTCGATAGTTTATTAATATTAGT | 645 | Sutcliffe et al., 1996 |
| *erm*(B) | F:GAAAAGRTACTCAACCAAATA R: AGTAACGGTACTTAAATTGTTTAC | 639 | Sutcliffe et al., 1996 |
| *erm*(C) | F:TCAAAACATAATATAGATAAA R: GCTAATATTGTTTAAATCGTCAAT | 642 | Sutcliffe et al., 1996 |
| *erm*(F) | F: GAGATCGGRCCAGGAAGC R: GTGTGCACCATCGCCTGA | 309 | Chen et al., 2007 |
| *mef*A | F: AGTATCATTAATCACTAGTGC R: TTCTTCTGGTACTAAAAGTGG | 348 | Sutcliffe et al., 1996 |
| *msr*A | F: GCAAATGGTGTAGGTAAGACAACT R: ATCATGTGATGTAAACAAAAT | 399 | Sutcliffe et al., 1996 |
| *tet*(M) | F: GTTAAATAGTGTTCTTGGAG R: CTAAGATATGGCTCTAACAA | 576 | Aarestrup et al., 2000 |
| *tet*(L) | F: CATTTGGTCTTATTGGATCG R: CAATATCACCAGAGCAGGCT | 456 | Aarestrup et al., 2000 |
| *tet* (S) | F: TGGAACGCCAGAGAGGTATT R: ACATAGACAAGCCGTTGACC | 660 | Aarestrup et al., 2000 |
| *tet* (K) | F: GATCAATTGTAGCTTTAGGTGAAGG R: TTTTGTTGATTTACCAGGTACCATT | 155 | Malhotra-Kumar et al. 2005 |
| *tet* (O) | F: AACTTAGGCATTCTGGCTCAC R: TCCCACTGTTCCATATCGTCA | 515 | Malhotra-Kumar et al. 2005 |
| *Cat*pIP 501-1 | F: GGATATGAAATTTATCCCTC R: CAATCATCTACCCTATGAAT | 486 | Aarestrup et al., 2000 |
| *catA7* | F: CCAGCAAACTACGTATAGCAT R: CGGTATGGTGTTTTCAGGTAT | 355 | Šeputiene et al. 2012 |
| *catA8* | F: GGATATGAACTGTATCCTGCT R: AATGAAACATGGTAACCATCAC | 461 | Šeputiene et al. 2012 |
| *catA9* | F: ATGGTTCGGGGAAATTGTTTC R: AAGCCAGTCATTAGGCCTATC | 278 | Šeputiene et al. 2012 |
| *cfr* | F: TGAAGTATAAAGCAGGTTGGGAGTCA R: ACCATATAATTGACCACAAGCAGC | 764 | Kehrenberg and Schwarz 2006 |
| *fex*A | F: GTACTTGTAGGTGCAATTACGGCTGA R: CGCATCTGAGTAGGACATAGCGTC | 1,272 | Kehrenberg and Schwarz 2006 |
| *lnu*B | F: CCTACCTATTGTTTGTGGAA R: ATAACGTTACTCTCCTATTC | 925 | Bozdogan et al., 1999 |
| *lnu*F | F: CACCATGCTTCAGCAGAAAATGATC R: TTACTTGTTGTGCGGCGTC | 1,200 | De Graef et al., 2007 |
| *tcr*B | F:CATCACGGTAGCTTTAAGGAGATTTTCR: ATAGAGGACTCCGCCACCATTG | 663 | Hasaman et al., 2006 |
| agg | F: AAGAAAAAGAAGTAGACCAAC R: AAACGGCAAGACAAGTAAATA | 1,553 | Eaton and Gasson, 2001 |
| gelE | F: ACCCCGTATCATTGGTTT R: ACGCATTGCTTTTCCATC | 419 | Eaton and Gasson, 2001 |
| cylM | F: CTGATGGAAAGAAGATAGTAT R: TGAGTTGGTCTGATTACATTT | 742 | Eaton and Gasson, 2001 |
| cylB | F: ATTCCTACCTATGTTCTGTTA R: AATAAACTCTTCTTTTCCAAC | 843 | Eaton and Gasson, 2001 |
| cylA | F: TGGATGATAGTGATAGGAAGT R: TCTACAGTAAATCTTTCGTCA | 517 | Eaton and Gasson, 2001 |
| esp | F: TTGCTAATGCTAGTCCACGACC R: GCGTCAACACTTGCATTGCCGAA | 933 | Eaton and Gasson, 2001 |
| efaAfs | F: GACAGACCCTCACGAATA R: AGTTCATCATGCTGTAGTA | 705 | Eaton and Gasson, 2001 |
| hyl | F: ACAGAAGAGCTGCAGGAAATG R: GACTGACGTCCAAGTTTCCAA | 276 | Zou et al., 2011 |
| cpd | F: TGGTGGGTTATTTTTCAATTC R: TACGGCTCTGGCTTACTA | 782 | Eaton and Gasson, 2001 |
| cob | F: AACATTCAGCAAACAAAGC R: TTGTCATAAAGAGTGGTCAT | 1,405 | Eaton and Gasson, 2001 |
| ccf | F: GGGAATTGAGTAGTGAAGAAG R: AGCCGCTAAAATCGGTAAAAT | 543 | Eaton and Gasson, 2001 |

1. Aarestrup FM, Agerso Y, Gerner-Smidt P, Madsen M, Jensen LB. Comparison of antimicrobial resistance phenotypes and resistance genes in *Enterococcus faecalis* and *Enterococcus faecium* from humans in the community, broilers and pigs in Denmark. Diagn. Microbiol. Infect Dis. 2000; 37: 127-137
2. Agersø Y, Pedersen AG, Aarestrup FM. Identification of *Tn5397*-like and *Tn916-like* transposons and diversity of the tetracycline resistance gene *tet*(M) in enterococci from humans, pigs and poultry. J Antimicrob Chemother. 2006; 57: 832–839
3. Bozdogan BL, Berrezouga MS, Kuo DA, Yurek KA, Farley BJ, Stockman R.et al. New resistance gene, *lin*B, conferring resistance to lincosamides by nucleotidylationin *Enterococcus faecium* HM1025. Antimicrob Agents Chemother. 1999; 43: 925–929.
4. Chen J, Yu Z, Michel FC, Wittum T, Morrison M. Development and application of real-time PCR assays for quantification of *erm* genes conferring resistance to macrolides-lincosamides-streptogramin B in livestock manure and manure management systems. Appl Environment Microbiol. 2007; 73: 4407–4416.
5. Clark NC, Olsvik O, Swenson JM, Spiegel CA, Tenover FC. Detection of a streptomycin/spectinomycin adenylyltransferase gene (*aadA*) in *Enterococcus faecalis.* Antimicrob Agents Chemother. 1999; 43: 157–160
6. De Graef EM, Decostere A, DeLeener E, Goossens H, Baele M, Haesebrouck F. Prevalence and mechanizm of resistance against macrolides, lincosamides, and streptogramins among *Enterococcus faecium* isolates from food-producing animals and hospital patients in Belgium. Microb Drug Resist*.* 2007; 13: 135–141.
7. Del Campo R, Tenorio C, Rubio C, Castillo J, Torres C, Gomez-Lus R. Aminoglycoside-modifying enzymes in high-level streptomycin and gentamicin resistant *Enterococcus* spp. in Spain. Int. J Antimicrob Agents. 2000; 15: 221–226.
8. Doherty NK, Trzcinski P, Pickerill P, Zawadzki C, Dowson G. Genetic diversity of the *tet*(M) gene in tetracycline-resistant clonal lineages of *Streptococcus pneumoniae.* Antimicrob Agents Chemother. 2000; 44: 2979–2984.
9. Eaton TJ, Gasson MJ. 2000 Molecular screening of E*enterococcus* virulence determinants and potential for genetic exchange between food and medical isolates Appl Environment Microbiol. 2000; 67: 1628–1635.
10. Hasman HI, Kempf B, Chidaine R, Cariolet AK, Ersbøll H, Houe HC. et al. Copper resistance in *Enterococcus faecium*, mediated by the *tcr*B gene, is selected by supplementation of pig feed with copper sulfate. Appl Environ Microbiol. 2006; 72: 5784–5789
11. Kehrenberg C, Schwarz C Distribution of florfenicol resistance genes *fexA* and *cfr* among chloramphenicol-resistant *Staphylococcus* isolates. Antimicrob Agents Chemother. 2006; 50: 1156–1163.
12. Zou L-K, Wang H-N, Zeng B, Li J-N, Li X-T, Zhang A-Y. et al. Erythromycin resistance and virulence genes in *Enterococcus faecalis* from swine in China. New Microbiologica. 2011; 34: 73-80.
13. Malhotra-Kumar S, Lammens C, Piessens J, Goossens H. Multiplex PCR for simultaneous detection of macrolide and tetracycline resistance determinants in streptococci. Antimicrob Agents Chemother. 2005; 49: 4798–4800.
14. Matsumura M, Katakura Y, Imanaka T, Aiba S. Enzymatic and nucleotide sequence studies of a kanamycin-inactivating enzyme encoded by a plazmid from thermophilic bacilli in comparison with that encoded by plasmid pUB110. J Bacteriol. 1984; 160: 413- 20.
15. Murphy E. Nucleotide sequence of a spectinomycin adenyltransferase AAD(9) determinant from *Staphylococcus* *aureus* and its relationship to AAD(3”)(9). Mol Gen Genet. 1985; 200: 33- 39.
16. Šeputiene V, Bogdaite A, Ružauskas M, Sužiedeliene E. Antibiotic resistance genes and virulence factors in *Enterococcus faecium* and *Enterococcus faecalis* from diseased farm animals: pigs, cattle and poultry. Polish J Vet Sci. 2012; 15: 431-438
17. Sutcliffe J, Grebe T, Tait-Kamradt A, Wondrack L. Detection of erythromycin-resistant determinants by PCR. Antimicrob Agents Chemother. 1996; 40: 2562–2566.
18. Vakulenko SB, Donabedian SM, Voskresenskiy AM, Zervos MJ, Lerner SA, Chow JW. Multiplex PCR for detection of aminoglycoside resistance genes in *Enterococci.* Antimicrob Agents Chemother. 2003; 47: 1423–1426
19. Van de Klundert JAM, Vliegenthart JS. PCR detection of genes for aminoglycoside-modifying enzymes. In: Persing, DH, TF. Smith, FC. Tenover, and TJ. White (eds), Diagnostic Molecular Microbiology. Principles and Applications. 1993. pp. 547–552. American Society for Microbiology, Washington D.C.
