# Supplementary material for: Characterization of Multidrug Resistant E. faecalis Strains from Pigs of Local Origin by ADSRRS-Fingerprinting and MALDI -TOF MS; Evaluation of the Compatibility of Methods Employed for Multidrug Resistance Analysis
Source: PLoS One. 2017 Jan 30;12(1):e0171160. doi: 10.1371/journal.pone.0171160 (PMC5279778; doi:10.1371/journal.pone.0171160)
Supplement: S3 Table — apotential genus-specific marker bpotential species-specific markers. (DOCX) [file pone.0171160.s005.docx]

S3 Table. The common peaks occured in all strains *E. faecalis* tested in this study

| peak m/z (the average) | standard deviation (SD) | confidence interwal (CI) |
| --- | --- | --- |
| 2214,76 | 0,588721 | 2214,805- 2214,715 |
| 3036,328 ^b^ | 0,554676 | 3036,504- 3036,152 |
| 3320,544 | 0,511627 | 3320,707-3320,381 |
| 3351,646 | 0,54156 | 3351,818-3351,474 |
| 3429,497 | 0,579847 | 3429,681-3429,313 |
| 3665,881 | 0,653224 | 3666, 673 -3665, 089 |
| 4411,587 | 0,658051 | 4411,796-4411,378 |
| 4428,443 ^a^ | 0,881878 | 4428,723- 4428,207 |
| 4556,098 | 0,742042 | 4556,862-4555, 334 |
| 4764,807 ^b^ | 0,903717 | 4765, 52-4764, 095 |
| 6077,325 ^b^ | 1,28654 | 6077,9159 -6076,734 |
| 6223,547 | 1,228267 | 6223,937-6223,156 |
| 6354,859 | 1,344089 | 6355,432 -6354,287 |
| 6396,836 | 1,748179 | 6397,392-6396,28 |
| 6669,426 | 1,142894 | 6669,789-6669,063 |
| 6857,914 ^b^ | 1,4208805 | 6857,462 -6857, 365 |
| 7019,937 | 1,781071 | 7020,503-7019,371 |
| 7328,15 | 1,577161 | 7328,751-7327,549 |
| 8103,351 | 1,891087 | 8103,952-8102,75 |
| 8822,0661 | 1,8472054 | 8822,653-8821,479 |
| 8875,92 | 1,963806 | 8876,545-8875,296 |
| 8949,698 | 2,136109 | 8950,377-8949,019 |
| 9104,4 ^b^ | 2,023291 | 9104,757 -9103,044 |
| 9522,606 | 2,169119 | 9523, 916 -9521,296 |
| 11112,82 | 2,536558 | 11113,62-11112,01 |

^a^potential genus-specific marker

^b^potential species-specific markers
